# Quadratic Concentration–Response Modeling and Molecular Docking of *Mespilodaphne quixos* (Lam.) Rohwer Essential Oil Against *Candida albicans*

**DOI:** 10.3390/molecules31111891

**Published:** 2026-06-01

**Authors:** Yasiel Arteaga-Crespo, Yudel García-Quintana, Yendrek Velásquez López, Matteo Radice, Mariana Magdalena Conforme-Garcia, Jannys Lizeth Rivera-Barreto, José Blanco-Salas, Reinier Abreu-Naranjo

**Affiliations:** 1 Departamento de Matemáticas y Ciencias Físicas, Faculta Ciencias de la Vida, Universidad Estatal Amazónica, Km 2 ½ Vía Puyo-Tena, Puyo 160150, Ecuador; yarteaga@uea.edu.ec (Y.A.-C.); mradice@uea.edu.ec (M.R.); jl.riverab@uea.edu.ec (J.L.R.-B.); 2 Departamento de Silvicultura y Producción Agrícola, Facultad de Ciencias de la Tierra, Universidad Estatal Amazónica, Km 2 ½ Vía Puyo-Tena, Puyo 160150, Ecuador; ygarcia@uea.edu.ec; 3 Departamento de Procesos Químicos, Alimentos y Biotecnología, Facultad de Ingeniería y Ciencias Aplicadas, Universidad Técnica de Manabí, Av. Urbina y Che Guevara, Portoviejo 130104, Ecuador; yendrek.velasquez@utm.edu.ec; 4 Laboratorio de Microbiología, Universidad Estatal Amazónica, Km 2 ½ Vía Puyo-Tena, Puyo 160150, Ecuador; mm.conformeg@uea.edu.ec; 5 Department of Vegetal Biology, Ecology and Earth Science, Faculty of Sciences, University of Extremadura, 06006 Badajoz, Spain; blanco_salas@unex.es

**Keywords:** *Mespilodaphne quixos*, *Candida albicans*, essential oil, antifungal, quadratic modeling, molecular docking

## Abstract

*Candida albicans* is an opportunistic fungal pathogen of clinical relevance, and plant-derived antifungal agents have attracted interest because of rising resistance to conventional drugs. This study aimed to characterize the chemical composition of *Mespilodaphne quixos* (Lam.) Rohwer essential oil (EO) by GC/MS, evaluate its in vitro antifungal activity against *C. albicans*, model its concentration-dependent response using one-factor quadratic polynomial modeling, and investigate the interactions of its constituents with selected fungal targets using molecular docking. Freshly collected leaves were subjected to steam distillation, then the EO was characterized using GC/MS. Antifungal activity was determined using the Kirby–Bauer disk diffusion method. A one-factor quadratic polynomial model was fitted to describe the inhibition halo diameter as a function of EO concentration. Moreover, 22 identified compounds were docked against 14-*α*-demethylase, Δ(14)-sterol reductase, and exo-*β*-(1,3)-glucanase. The EO was mainly composed of (*E*)-cinnamaldehyde (47.2%), caryophyllene (10.8%), and *α*-humulene (5.37%). The EO reached an inhibitory capacity of 87.3% relative to ketoconazole. The quadratic model showed good predictive performance. Molecular docking revealed favorable affinities for several sesquiterpenes present in *M. quixos* essential oil: *α*-copaene showed the best interaction profile against 14-*α*-demethylase and Δ(14)-sterol reductase, whereas α-guaiene and spathulenol performed best against exo-*β*-(1,3)-glucanase. These findings provide preliminary in vitro and in silico evidence supporting the antifungal activity of *M. quixos* EO.

## 1. Introduction

In recent decades, infections due to *Candida albicans* have increased to the point that the World Health Organization (WHO) has named it a “critical priority pathogen.” Studies aimed at combating its spread have necessarily increased since this human pathogen is one of the leading causes of invasive candidiasis and deaths in immunocompromised patients. The search for new potential bioactive compounds depends on various factors, such as interactions with the immune system, antifungal resistance, and the biological characteristics of *C. albicans* itself. Considering the differing hygienic–sanitary conditions and the efficiency of hospital systems across the world, it is estimated that the mortality rates associated with invasive candidiasis can occur in a range that includes 40 to 75%. At a global level, it has been calculated that approximately 250,000–700,000 systemic infections occur each year, of which 50,000–100,000 are fatal [1,2].

The main virulence factors in candidiasis include the pathogen’s ability to adhere and form biofilms, which hinder antifungal penetration and increase tolerance. Enzyme production, phenotypic switching, and antifungal resistance mechanisms further contribute to the persistence and pathogenicity of *C. albicans*. This enables *C. albicans* to proliferate on the skin, in the gastrointestinal tract, and on oral and vaginal mucous membranes. The therapeutic approach to candidiasis requires the administration of synthetic antimycotics (itraconazole, clotrimazole, fluconazole, and ketoconazole), but numerous studies have investigated the integration of these treatments with substances of plant origin, such as essential oils (EOs) and hydrolates, which have been shown to be effective in assisting pharmacological treatments [3,4,5].

EOs and other plant derivatives appear to be effective against clinical strains of *C. albicans*, boosting the immune system and inhibiting the formation and expansion of *C. albicans* biofilm [6,7]. Finally, the treatment of candidiasis is frequently hindered by relapses or the emergence of strains resistant to conventional therapies [4,8]. Consequently, there is a compelling need to explore novel experimental strategies, particularly those investigating the synergistic effects of antifungals combined with essential oils or their bioactive components, such as eugenol and citral. Such approaches may significantly enhance therapeutic efficacy [9,10]. Against this background, essential oils remain a key source of naturally occurring bioactive compounds and represent a frontier in the investigation of *C. albicans*. Furthermore, as the essential oil of *M. quixos* has been scarcely investigated, the aim of this study was to partially bridge the knowledge gap regarding its biological activity.

With this in mind, the present study focuses on the *in vitro* antifungal activity of the EO of *Mespilodaphne quixos* (Lam.) Rohwer (Syn. *Ocotea quixos* (Lam.) Kosterm), which has been the focus of research pertaining to its essential oil and merits further scrutiny concerning both its phytochemical characterization and biological properties [11,12,13]. *M. quixos* belongs to the Lauraceae family, which comprises 55 genera and is widespread throughout the planet [14]. *M. quixos* is a native tree present in the Andean and Amazon regions of Ecuador and is commonly called “*ishpinko*” or “*ishpingo*” as well as “*canelo*” or “*canela del oriente*.” The latter two names derive from the fact that various botanical parts of the species have an aroma similar to that of the species *Cinnamomum verum* J.Presl, whose common name in Spanish is precisely “*canela*.” *M. quixos* trees grow up to 30 m tall and have a large, rounded trunk. Their bark is smooth, and their leaves are simple, alternate, spirally arranged, oblong, acuminate, glabrous, entire, leathery, and green, measuring 10 to 15 cm long and 4 to 5 cm wide. Their flowers are axillary capsules with a semi-woody calyx. They produce a reddish, ellipsoid drupe, 1.5–2 cm long, on a cap-like cupule with a double margin. Their seeds are approximately 2 cm in diameter [15]. *M. quixos* essential oils can be extracted from the bark, leaves, and calyxes and have been studied for their antimicrobial, antioxidant, anti-inflammatory, and larvicidal activity. Among the oil’s compounds, the following stand out: (*E*)-Methyl cinnamate, (*E*)-Caryophyllene, (*E*)-Cinnamyl acetate, (*E*)-Cinnamaldehyde, 1,8-cineole, sabinene, *α*-pinene, and terpinen-4-ol [12,13,16].

In recent years, several experimental, statistical, and in silico tools have been applied to evaluate and predict biological responses associated with natural products and their bioactive constituents [17,18]. Among these approaches, polynomial concentration–response modeling and molecular docking are particularly relevant in the context of antifungal research [19]. Quadratic polynomial models can be used to describe the effect of experimental conditions, estimate concentration-dependent biological responses, and support the interpretation of antifungal performance [20]. Molecular docking, in turn, has been extensively applied to estimate the interactions between bioactive compounds and specific molecular targets, thus providing mechanistic support for experimentally observed biological effects [21]. Building on this approach, the integration of in vitro evidence with computational frameworks has been applied in analogous studies to support the biological relevance of natural compounds as antifungal candidates against *C. albicans* [22,23], underscoring the relevance of combined experimental and computational strategies in the early characterization of bioactive plant extracts.

Therefore, this study aimed to characterize the chemical composition of *M. quixos* (Lam.) Rohwer essential oil (EO) by GC/MS, evaluate its in vitro antifungal activity against *C. albicans*, model its concentration-dependent response using one-factor quadratic polynomial modeling, and investigate the interactions of its constituents with selected fungal targets using molecular docking.

## 2. Results

### 2.1. Extraction Yield of EO Obtained from M. quixos

The essential oil from fresh *M. quixos* leaves was obtained by steam distillation. The oil had a pale-yellow color and a characteristic cinnamon-like aromatic odor. The yield was 0.32 mL/100 g fresh weight (*v*/*w*) calculated from the total EO volume recovered across the independent extractions and the total fresh leaf mass processed. This value corresponds to extractions carried out under the same operating conditions.

### 2.2. Chemical Characterization of the EO Obtained from M. quixos

**Figure 1:**
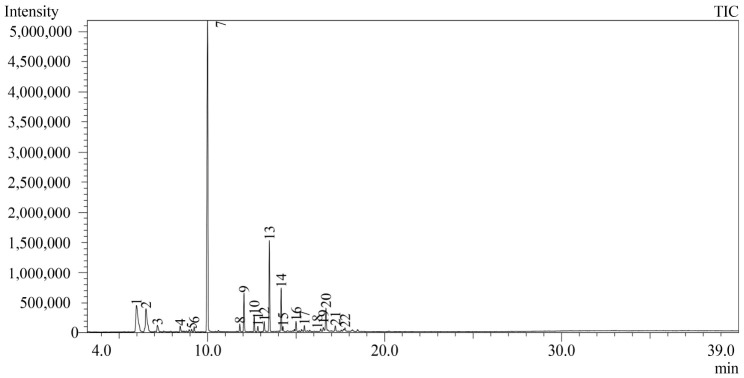
Chromatogram of the *M. quixos* EO.

The GC/MS analysis of the EO obtained from *M. quixos* revealed a total of 22 compounds, accounting for approximately 99.9% of the total chromatogram area (Figure 1).

The chemical profile of *M. quixos* EO was characterized by the predominance of phenylpropanoid derivatives, mainly represented by (*E*)-cinnamaldehyde (47.2%), followed by methyl cinnamate (4.63%), (*E*)-cinnamyl acetate (1.16%), benzenepropanal (0.81%), (*Z*)-cinnamaldehyde (0.61%), and *cis*-isomethyleugenol (0.58%). Sesquiterpenes constituted the most diverse chemical group, including major components such as caryophyllene (10.8%), *α*-humulene (5.37%), 14-hydroxycaryophyllene (2.98%), *α*-copaene (1.93%), and bicyclogermacrene (1.33%), together with other minor hydrocarbon and oxygenated sesquiterpenes. Monoterpenes were represented mainly by *α*-pinene (9.63%) and *β*-pinene (7.08%), with lower proportions of *β*-terpinyl acetate (1.36%) and *α*-terpineol (0.31%). Overall, these results indicate that *M. quixos* EO is defined by a phenylpropanoid-rich profile accompanied by a chemically diverse sesquiterpene fraction. The complete list of tentatively identified compounds, including retention times and relative percentages, is presented in Table 1.

This chemical diversity highlights the complex phytochemical profile of *M. quixos* EO, characterized by a predominance of phenylpropanoids and a broad array of terpenoids. The coexistence of structurally diverse metabolites may contribute to a broad-spectrum mechanism of action, supporting the potential of this essential oil for subsequent bioactivity evaluations.

### 2.3. Antifungal Activity of M. quixos EO against C. albicans

The antifungal activity of *M. quixos* EO against *C. albicans* was assessed using the Kirby–Bauer disk diffusion method. The predicted inhibition halo diameters were in close agreement with the experimental measurements, indicating a good fit of the model to the observed data. The observed and predicted values for each experimental run are presented in Table 2.

**Figure 2:**
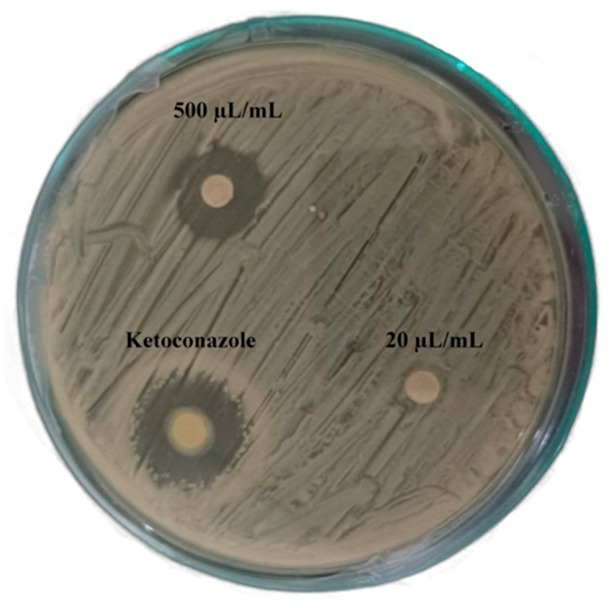
Inhibition halos of *C. albicans* observed in the experimental design with *M. quixos* EO at 20 µL/mL (low concentration), 500 µL/mL (high concentration), and the ketoconazole (positive control).

The inhibition halos obtained in the experimental design ranged from 5.00 mm to 17.0 mm (Table 2), reflecting a proportional increase with increasing concentrations of *M. quixos* EO. For the lowest concentrations (20 µL/mL), the inhibition halos were 5.5 ± 0.5 mm, indicating low antifungal activity. At 260 µL/mL and above, more consistent responses were observed, with an average of 12.7 ± 0.2 mm, indicating an improvement in inhibitory capacity. The highest inhibition was achieved at a concentration of 500 µL/mL, with a maximum value of 17.0 mm (Run 9, Table 2) and a mean of 16.0 ± 1.0 mm overall for this concentration. The inhibition halos for the lowest and highest concentrations tested, as well as the positive control, are shown in Figure 2.

These results demonstrate the antifungal potential of the essential oil against *C. albicans* and justify its inclusion in further validation and therapeutic application studies.

### 2.4. Modeling the Antifungal Properties of M. quixos EO

The results of the ANOVA (Table 3) for the quadratic model showed that both the overall model and each of its terms were significant, indicating a strong statistical relationship between the concentration of the essential oil and the inhibition halo generated against *C. albicans*. The non-significant lack of fit (*p* = 0.3201) suggests that deviations between observed and predicted values are attributable to experimental error rather than model inadequacy. In addition, the corrected total sum of squares (Cor Total = 206) reflects the total variability in the response, providing a basis for assessing the model’s capacity to explain the dispersion observed in the experimental data. These findings support the suitability of the quadratic model for accurately representing the system under study.

Regarding model fit, the coefficient of determination (R^2^) was 0.978, with an adjusted R^2^ of 0.974 and a predicted R^2^ of 0.961. These values indicate that the model has an excellent explanatory capacity and that there is a high consistency between fitting and predictive ability. The difference between the adjusted and predicted R^2^ values was less than 0.2, indicating strong internal agreement. Moreover, the residual standard deviation was 0.615, and the model’s coefficient of variation was 5.29%, demonstrating the precision of the estimates.

**Figure 3:**
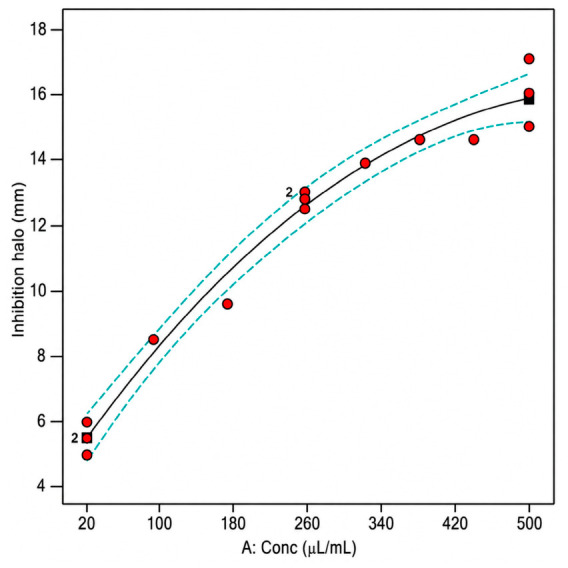
Quadratic concentration–response model for the inhibition halo diameter as a function of *M. quixos* EO concentration. Red circles represent the experimental inhibition halo values, the black square represents the predicted model value, the solid black line represents the fitted quadratic model, and the dashed lines represent the model uncertainty bands generated by the software. Conc = concentration.

Figure 3 shows the relationship between the concentration of *M. quixos* EO and the inhibition halo diameter, calculated using the fitted quadratic model. The curve shows a non-linear behavior, with an upward trend that begins to stabilize around 400 µL/mL, reaching a maximum inhibitory effect of approximately 16 mm. This behavior indicates that increasing the concentration of essential oil improves the antifungal activity up to an inhibition value at which further increases do not produce significant improvements in the response variable.

The model accurately predicts the observed values across the entire experimental range (20–500 µL/mL), confirming its usefulness as a tool for estimating antifungal response according to concentration. The final quadratic equation, expressed in real units, allows for the estimation of the inhibition halo (Y) in mm based on the essential oil concentration (A) in µL/mL, and can be expressed as follows.

Y = 4.60 + 0.03773 × A − 0.00003 × A^2^ (1)

### 2.5. Target Fishing and Homology Identification

A computational target fishing analysis was performed by integrating the Similarity Ensemble Approach (SEA) and SwissTargetPrediction platforms, followed by homology-based mapping against *C. albicans.* The initial screening identified 59 potential protein candidates associated with essential metabolic pathways in *C. albicans*. The complete target fishing and homology mapping output is provided in Supplementary Materials S1, including the prioritized targets, the consolidated list of unique *C. albicans* homologous candidates, and the complete RefSeq target identification output (Tables S1–S3). Based on sequence identity, query coverage, structural availability, and biological relevance, three enzymatic targets were prioritized for molecular docking: 14-*α*-demethylase (CYP51), Δ(14)-sterol reductase, and exo-*β*-(1,3)-glucanase. These findings are summarized in Table 4, with target prioritization based on their roles in ergosterol biosynthesis and fungal cell wall remodeling.

### 2.6. Molecular Docking and Interaction Network Analysis

Molecular docking was performed for the 22 compounds identified in the essential oil of *M. quixos* against three fungal targets: 14-*α*-demethylase, Δ(14)-sterol reductase, and exo-*β*-(1,3)-glucanase. The binding energies ranged from −5.8 to −8.2 kcal/mol across all ligand–target combinations, and the corresponding affinity values are summarized in Table 5.

Among the essential oil constituents, *α*-copaene showed the most favorable binding energy toward 14-*α*-demethylase (−7.9 kcal/mol) and Δ(14)-sterol reductase (−7.1 kcal/mol), whereas *α*-guaiene and spathulenol showed the best values against exo-*β*-(1,3)-glucanase (−8.2 kcal/mol). Ketoconazole showed stronger binding energies for all three evaluated targets, with values of −10.8 kcal/mol for 14-*α*-demethylase, −9.5 kcal/mol for Δ(14)-sterol reductase, and −9.2 kcal/mol for exo-*β*-(1,3)-glucanase. The binding poses and interaction patterns of the selected compounds are presented in the corresponding figures.

**Figure 4:**
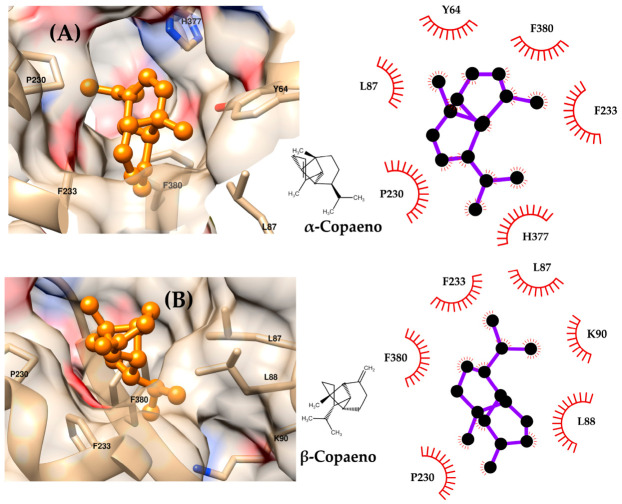
Binding modes and interaction profiles of *α*-copaene and *β*-copaene with 14-*α*-demethylase (PDB ID: 5TZ1). (**A**) Interaction profile of *α*-copaene. (**B**) Interaction profile of *β*-copaene, showing stabilization through hydrophobic contacts (red arcs).

As shown in Figure 4, *α*-copaene and *β*-copaene exhibited a similar binding pattern within the active site of 14-*α*-demethylase. Both isomers were accommodated within the hydrophobic cavity, with binding poses mainly stabilized by steric complementarity and non-polar interactions with aromatic and aliphatic residues. These results indicate similar predicted binding modes for both sesquiterpenes within the CYP51 binding pocket.

**Figure 5:**
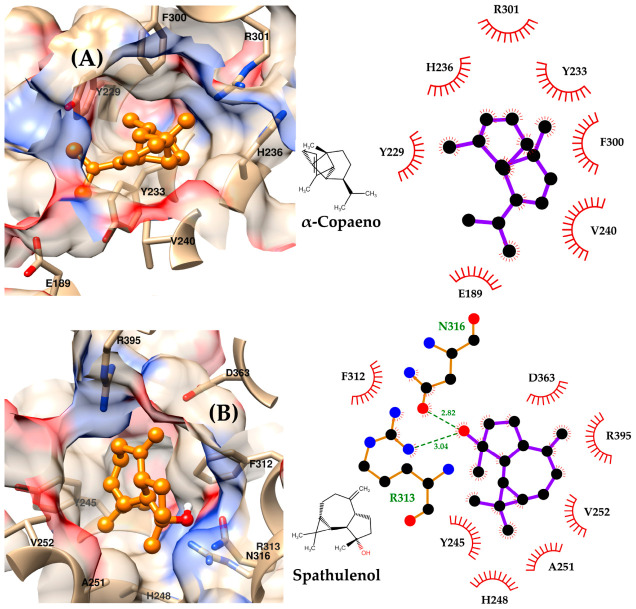
Binding modes and interactions of *α*-copaene and spathulenol with Δ(14)-sterol reductase (PDB ID: 4QUV). (**A**) Interaction profile of α-copaene, mainly showing hydrophobic contacts. (**B**) Interaction profile of spathulenol, showing hydrophobic contacts and conventional hydrogen bonds. In the 3D representations, the ligand is shown in orange within the protein binding pocket. In the 2D diagrams, black circles represent ligand atoms, red arcs indicate hydrophobic contacts with amino acid residues, green dashed lines indicate conventional hydrogen bonds, and green numbers indicate hydrogen bond distances in Å.

The analysis of the docking poses for Δ(14)-sterol reductase revealed different interaction patterns for *α*-copaene and spathulenol (Figure 5). *α*-Copaene interacted mainly through hydrophobic contacts within the aromatic-rich catalytic site. In contrast, spathulenol showed a dual interaction profile, combining hydrophobic packing with two conventional hydrogen bonds at 2.82 Å and 3.04 Å, favored by the presence of its hydroxyl group. These results indicate that spathulenol forms additional polar interactions compared with the predominantly hydrophobic binding pattern observed for *α*-copaene.

**Figure 6:**
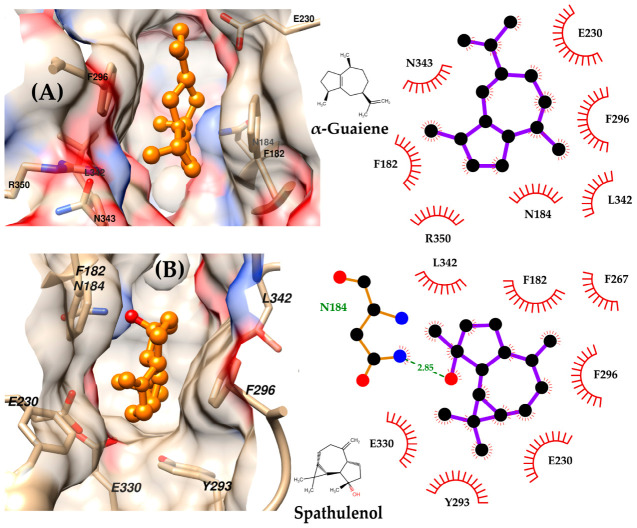
Binding modes and interactions of *α*-guaiene and spathulenol with *exo*-*β*-(1,3)-glucanase (PDB ID: 1EQC). (**A**) Interaction profile of *α*-guaiene, mainly showing hydrophobic contacts. (**B**) Interaction profile of spathulenol, showing hydrophobic contacts and one conventional hydrogen bond. In the 3D representations, the ligand is shown in orange within the protein binding pocket. In the 2D diagrams, black circles represent ligand atoms, red arcs indicate hydrophobic contacts with amino acid residues, the green dashed line indicates a conventional hydrogen bond, and the green number indicates the hydrogen bond distance in Å.

For exo-*β*-(1,3)-glucanase, the computational analysis revealed different interaction patterns for *α*-guaiene and spathulenol (Figure 6). *α*-Guaiene mainly established hydrophobic contacts within the catalytic pocket. In contrast, spathulenol displayed a more diverse interaction profile, combining hydrophobic packing with a conventional hydrogen bond between its hydroxyl group and N184 at 2.85 Å. Overall, both ligands were accommodated within the active site, with spathulenol showing an additional polar interaction compared with the predominantly non-polar interaction pattern observed for *α*-guaiene.

The molecular docking results showed that several sesquiterpenes from the essential oil of *M. quixos* exhibited favorable binding affinities toward the three fungal targets evaluated. *α*-Copaene showed the most favorable interaction profile against 14-*α*-demethylase and Δ(14)-sterol reductase, whereas *α*-guaiene and spathulenol stood out against exo-*β*-(1,3)-glucanase. Overall, these findings provide preliminary in silico support for the possible contribution of these compounds to the antifungal activity observed experimentally.

## 3. Discussion

The essential oil yield obtained from fresh *M. quixos* leaves (0.32 mL/100 g fresh weight) was slightly higher than the 0.24% reported by Arteaga-Crespo et al. [24] for *M. quixos* and much higher than that obtained by Gilardoni et al. [11] (a weight-based yield of 1.52%). Yet, the yield reported for the present research was lower than the 0.37, 0.51, and 0.96 mL/100 g values reported for *Ocotea leptobotra* (Ruiz & Pav.) Mez, *Ocotea puberula* (Rich.) Nees, and *Ocotea odorifera* (Vell.) Rohwer, respectively, by Gil et al. [25] and Mezzomo et al. [26], all belonging to the same family (Lauraceae). These differences in yield can be attributed to various factors, including genetic variability among species, agroecological cultivation conditions, the physiological state of the plants at the time of harvest, and specific distillation parameters such as process duration and steam flow rate [27,28]. Additionally, in the case of *M. quixos*, the relatively low yield may be related to a lower concentration of volatile compounds in fresh leaves, as other studies have reported higher essential oil yields when leaves were subjected to drying treatments prior to extraction [29].

The chemical diversity observed, with 22 tentatively identified compounds belonging to different structural groups, reflects the metabolic complexity within the Lauraceae family. Discrepancies in the chemical profile of the essential oil are evident in the literature. Gilardoni et al. [11] reported a prevalence of (*E*)-cinnamyl acetate (46.0–50.4%), whereas Sosa et al. [12] found (*E*)-methyl cinnamate to be the major component, albeit at a lower concentration (19.3%).

The chemical profile identified in the essential oil of *M. quixos* presents distinctive features that set it apart from other species within the Lauraceae family, including those historically grouped under the genus *Ocotea*. Most species within this taxonomic group are characterized by volatile profiles dominated by monoterpenes and sesquiterpenes, as reported for *O. leptobotra* [25], *O. puberula* [26,30], and *O. odorifera* [31], where compounds such as *β*-caryophyllene, germacrene D, and *α*-humulene constitute the main components. However, other species within the Lauraceae family, such as those belonging to the genus *Cinnamomum*, have also been reported to possess volatile profiles dominated by phenylpropanoids. In a study conducted by Rawat et al. [32], the essential oil of *Cinnamomum tamala* (Buch.-Ham.) T.Nees & C.H.Eberm. was found to contain (*E*)-cinnamaldehyde (21.04%) and (*E*)-cinnamyl acetate (54.49%) as major components, similar to what was found in this study.

This type of differentiation in chemical composition has been documented even within the same species. For instance, Saha et al. [33] observed markedly distinct chemical profiles in essential oils extracted from *C. verum* collected in different geographical regions of India. The essential oils from leaves collected in Kharagpur exhibited a profile dominated by phenylpropanoids (81.98%), whereas those from Mamit were characterized by a predominance of monoterpenoids (48.42%), with eugenol and linalool as the principal compounds, respectively. Despite the absence of significant structural differences in the secretory tissues, the variation in chemical composition enabled the classification of these populations into distinct chemotypes. Their study concluded that environmental and geographical factors can significantly influence the phytochemical profile of species within the Lauraceae family, which could also help explain the unique chemical composition of *M. quixos*. These findings highlight the importance of chemotaxonomic and ecological factors when evaluating essential oil profiles. The compositional uniqueness of *M. quixos*, characterized by its phenylpropanoid-rich profile, reinforces the need for further investigations aimed at understanding the ecological drivers and biosynthetic pathways.

The antifungal activity of *M. quixos* EO against *C. albicans* exhibited a behavior characterized by an increase in the inhibition halo diameter proportional to the rising concentration of the oil. A previous study performed by Sosa et al. [12] uncovered a minimum inhibitory concentration (MIC) value of 273.69 µg/mL. This pattern is consistent with that reported in other in vitro studies of essential oils, where the accumulation of bioactive compounds in the medium inhibits the growth of the pathogenic fungus [34,35]. Furthermore, a slight tendency towards stabilization was observed at concentrations above 400 µL/mL, suggesting a possible saturation threshold in the inhibitory capacity of the essential oil. This phenomenon could be related to the saturation of specific target sites, either at the membrane level or in enzymatic systems of *C. albicans*, thus limiting the inhibitory efficacy once a critical concentration of the oil’s main active compounds is reached [36,37].

The main findings concerning the MIC, minimum fungicidal concentration (MFC), and biofilm inhibition in *C. albicans* have been discussed extensively [6,7,9]. Furthermore, evidence has emerged regarding the ability of these compounds to enhance the intracellular killing activity of human polymorphonuclear leukocytes (PMNs) against *C. albicans* while reducing biofilm formation and viability. As reported by Shahina and Dahms [9], certain key components of essential oils—such as 1,8-cineole, α-pinene, eugenol, and citral—appear capable of delocalizing Kar3p (kinesin motor protein Kar3), thereby damaging microtubules and inducing the formation of pseudopodia, which ultimately limits biofilm formation.

The highest inhibition halo produced by *M. quixos* EO was 17.0 mm at 500 µL/mL, corresponding to 87.3% of the inhibition halo produced by ketoconazole under the same assay conditions. This comparison is relevant because ketoconazole remains a standard azole reference in antifungal susceptibility assays against *Candida* spp., and prior evidence suggests that its activity may be potentiated in association with natural phenolic compounds [38]. Although cross-study comparisons must be interpreted with caution due to differences in assay design, strain susceptibility, and response criteria, this result places *M. quixos* EO within the range of biologically active plant-derived oils reported against *C. albicans*.

In this regard, essential oil from *Origanum majorana* L. exhibited marked activity against both planktonic and biofilm-forming *C. albicans*, with IC_50_ and IC_90_ values of ≤ 0.5 µg/mL and strong inhibition of germ-tube formation, whereas *Eugenia uniflora* L. oil required substantially higher concentrations and showed indifferent or antagonistic interactions with fluconazole [35,39]. Against this background, the activity recorded for *M. quixos* EO reinforces its relevance as a promising Amazonian source of anticandidal metabolites and provides a coherent basis for relating the observed biological response to its chemical composition.

However, the antifungal activity depends on both the presence and concentration of bioactive compounds in the essential oil [40]. In the case of *M. quixos* EO, this activity could be attributed to the high proportion of phenylpropanoids, among which (*E*)-cinnamaldehyde was identified as the most abundant component. This compound has been widely documented for its ability to alter the permeability of the plasma membrane and organelles, as well as to disrupt the energy metabolism of pathogenic fungi, compromising their structural integrity and causing a loss of essential intracellular components [41,42,43]. In addition, it has been suggested that (*E*)-cinnamaldehyde interferes with key metabolic pathways, such as ergosterol biosynthesis and oxidative enzyme activity. Its inhibitory effect against *Aspergillus niger* DTZ-12 has also been reported to be significantly stronger than that of other common antifungal agents such as eugenol, carvacrol, and linalool [44]. The presence of sesquiterpenes such as caryophyllene, *α*-humulene, and humulene epoxide II could also contribute synergistically to the inhibitory effect by inducing oxidative stress or altering cell membrane-associated functions [45]. The phytochemical composition of *M. quixos* EO therefore suggests a multifactorial effect, with different mechanisms of action acting in a complementary manner to inhibit *C. albicans* growth, which may explain the high relative efficacy observed in this study.

Nevertheless, it should be noted that these evaluations were conducted under in vitro conditions; therefore, further studies should validate these findings in more complex models that reflect biological activity under real physiological conditions, as well as explore possible synergistic mechanisms with conventional antifungal agents.

On the other hand, the quadratic model employed adequately described the relationship between the concentration of *M. quixos* EO and the antifungal response against *C. albicans*. This behavior is common in biological systems, as increasing the concentration of an active agent may lead to saturation thresholds or points of maximum inhibition, resulting in a parabolic or curvilinear response [39]. The quadratic model has been widely used in studies evaluating the efficacy of natural compounds, including essential oils, plant extracts, or pure metabolites, where the chemical complexity of the matrix can induce non-linear responses against pathogenic microorganisms [46,47,48].

The high values of R^2^, adjusted R^2^, and predicted R^2^ obtained in this study support the statistical robustness of the applied quadratic model. The closeness between these coefficients indicates that the model not only fits the observed experimental data well but also exhibits reliable predictive capacity within the evaluated range. The minimal difference between the adjusted and predicted values demonstrates that the inclusion of the quadratic term significantly contributed to the model’s fit without compromising its stability [49].

The developed quadratic model constitutes a practical tool for accurately estimating the concentrations of *M. quixos* EO that produce an inhibitory effect against *C. albicans* without the need for additional experimental trials within the evaluated range [40]. This predictive capability is particularly valuable during the formulation stages of biofungicides, where it is essential to optimize the effective dose while minimizing raw material usage. Furthermore, the model may serve as a basis for the design of factorial studies or validation experiments that incorporate additional factors, such as fungal strain type or variable environmental conditions [50]. Although the model has demonstrated robustness within the established experimental framework, it would be advisable to assess its performance under other biological conditions or against different *Candida* strains and other phytopathogens, in order to broaden its applicability and confirm its predictive value in wider contexts [51].

The target fishing and homology mapping analysis supported the prioritization of 14-*α*-demethylase (CYP51), Δ(14)-sterol reductase, and exo-*β*-(1,3)-glucanase as relevant fungal targets for docking analysis. These proteins are associated with key physiological processes in *C. albicans*, particularly ergosterol biosynthesis and cell wall remodeling. CYP51 and Δ(14)-sterol reductase participate in sterol metabolism, which is essential for maintaining fungal membrane integrity and fluidity. Exo-*β*-(1,3)-glucanase is related to *β*-glucan metabolism and cell wall remodeling, a process of interest because the fungal cell wall is absent in mammalian cells. The selection of these targets is also consistent with previous docking-based anticandidal studies in which fungal proteins associated with glucan metabolism and ergosterol biosynthesis were evaluated as molecular targets for natural antifungal compounds [52].

The molecular docking results provided preliminary structural insight for the experimental antifungal activity. The interaction patterns identified across the evaluated complexes appeared to be influenced by the intrinsic physicochemical properties of the ligands. Sesquiterpene hydrocarbons such as *α*-copaene, *β*-copaene, and *α*-guaiene lack heteroatoms, limiting their ability to establish directional interactions such as hydrogen bonds. As a result, their predicted binding profiles were mainly associated by non-specific forces, particularly London dispersion and hydrophobic contacts within non-polar regions of the target proteins.

For lanosterol 14-*α*-demethylase (CYP51), the docking poses showed that both copaene isomers, *α*-copaene and *β*-copaene, were accommodated within a predominantly hydrophobic cavity (Figure 4). Their predicted binding modes involved close contacts with aromatic and aliphatic residues, including phenylalanine (F), tyrosine (Y), and leucine (L). This interaction pattern is consistent with the binding behavior expected for non-polar terpenes and with the relevance of CYP51 as a key enzyme in ergosterol biosynthesis [19,53].

A different interaction pattern was observed for Δ(14)-sterol reductase and *exo*-*β*-(1,3)-glucanase. In these targets, ligand recognition appeared to be influenced by specific structural features of the sesquiterpenes. While *α*-copaene and *α*-guaiene showed interaction profiles dominated by hydrophobic contacts, spathulenol introduced an additional polar component due to the presence of its hydroxyl group. This group was associated with localized contacts and hydrogen bonding with residues such as R313 and N316 in Δ(14)-sterol reductase and N184 in *exo*-*β*-(1,3)-glucanase, suggesting a possible contribution to complex stabilization.

For Δ(14)-sterol reductase, the binding pattern showed differences between *α*-copaene and spathulenol (Figure 5). While *α*-copaene was mainly associated with hydrophobic contacts within an aromatic-rich environment, spathulenol showed additional polar interactions due to the presence of its hydroxyl group. This functional group was associated with hydrogen bonding involving residues such as R313 and N316, which may contribute to a more stable predicted binding arrangement. Similar behavior has been reported in molecular dynamics studies, where the hydroxyl group of spathulenol was associated with increased conformational stability of the ligand–protein complex [54].

The exo-*β*-(1,3)-glucanase models reflect a comparable trend. *α*-Guaiene was mainly associated with hydrophobic contacts, whereas spathulenol showed additional polar interactions involving N184 (Figure 6) [55]. These findings indicate that structural differences among sesquiterpenes may influence their predicted interaction profiles, in agreement with Khan et al. [56] for other plant-derived sesquiterpenes. Overall, these results suggest that several sesquiterpenes in the essential oil may contribute to the antifungal effect through interactions with relevant molecular targets of *C. albicans*. However, these observations should be considered as preliminary mechanistic insights rather than direct evidence of enzymatic inhibition.

Despite these structurally favorable arrangements, it is important to acknowledge that docking scoring functions provide only approximate estimates and may not fully capture the dynamic behavior of protein–ligand systems or the influence of solvent effects [57,58]. Considering these limitations, the interaction patterns observed in this study are consistent with a possible multi-target mode of action rather than strong inhibition of a single enzyme. The data suggest that moderate but complementary interactions may occur across different proteins involved in key physiological processes of *C. albicans*. In this context, *α*-copaene showed a preferential predicted interaction with 14-*α*-demethylase and Δ(14)-sterol reductase, whereas *α*-guaiene and spathulenol showed comparatively favorable predicted binding within the active site of exo-*β*-(1,3)-glucanase.

Notably, while (*E*)-cinnamaldehyde is the dominant constituent of *M. quixos* EO (47.2%), the highest binding affinities were predominantly observed for sesquiterpenes such as *α*-copaene and *α*-guaiene. This apparent discrepancy indicates that antifungal activity may not depend exclusively on the compounds with the highest predicted binding affinities. Instead, the biological response may involve the combined contribution of abundant compounds with moderate affinity, such as (*E*)-cinnamaldehyde, and less abundant sesquiterpenes with more favorable shape complementarity within the selected fungal targets. Therefore, the observed antifungal effect is likely multifactorial and may involve complementary interactions among phenylpropanoids and sesquiterpenes present in the EO. From a drug discovery perspective, these naturally occurring scaffolds may represent useful starting points for future optimization studies, although additional experimental validation is required [59].

Future studies should complement the docking results with molecular dynamics simulations and protonation-state analyses to evaluate the influence of pH, temperature, solvent effects, and receptor flexibility on the stability of the predicted ligand–target complexes. ADMET prediction should also be included to better characterize the pharmacokinetic behavior, drug likeness, and potential toxicity of the predominant and best-docked EO constituents. In addition, the biological evaluation of *M. quixos* should be expanded by comparing the antifungal activity of different extracts, fractions, and plant organs, including leaves, bark, and calyces. The EO and its major constituents should also be assessed against a broader panel of fungal strains, including clinical and resistant isolates of *Candida* spp. and other medically relevant fungi. Finally, toxicity and selectivity assays in mammalian cell models, followed by in vivo studies, would help define the safety profile, antifungal efficacy, and therapeutic relevance of this EO.

## 4. Materials and Methods

### 4.1. Samples

The leaves of *M. quixos* were collected during the early morning hours in December 2024. The samples were obtained from ten spatially separated adult trees located on the main campus of the Universidad Estatal Amazónica in Puyo, Pastaza Province, Ecuador (1°28′00.1′’ S, 77°59′49.0″ W). Sampling was conducted following a random design to ensure the representativeness of the samples. Botanical identification was carried out with the assistance of Dr. Diego Gutiérrez del Pozo at the Herbarium of the Universidad Estatal Amazónica (ECUAMZ).

### 4.2. EO Extraction

The essential oil from *M. quixos* leaves was extracted using steam distillation. Fresh leaves were placed in a FIGMAY laboratory-scale essential oil extractor (model: FIGMAY S.R.L. laboratory scale, Córdoba, Argentina), following the procedure reported by Berrú et al. [60] with minor adaptations. For each extraction, approximately 500 g of fresh leaves were placed in the stainless-steel plant basket of the extractor. The apparatus consisted of a borosilicate glass boiler, a heating system with electrical resistance, a borosilicate glass condenser, a graduated collector connected to the distillation system, and a constant-level water supply system. The equipment was connected directly to the running-water network; the water flow entered through the condenser and then fed the boiler through the constant-level system. During operation, the water level was maintained above the electrical resistance and the safety level sensors. Steam distillation was performed via the continuous generation of water vapor at approximately 100 °C. The process was stopped after 40 min, when no further increase in the recovered oil volume was observed. A total of eight independent distillations were carried out under the same operating conditions. The collected EO was allowed to cool to room temperature (~20 °C), separated from the aqueous phase, dried over anhydrous sodium sulfate, filtered, and stored in sealed amber vials at 4 °C until analysis. The EO volume was read directly from the graduated collector after phase separation, and the extraction yield was expressed as mL of EO per 100 g of fresh leaves (mL/100 g FW, *v*/*w*).

### 4.3. Gas Chromatography–Mass Spectrometry Analysis

The essential oil from fresh *M. quixos* leaves was analyzed using gas chromatography–mass spectrometry (GC/MS) on a Shimadzu QP2020 NX system (Shimadzu Europe, Duisburg, Germany), following the method described by Berrú et al. [60] with slight modifications. In brief, 250 µL of *M. quixos* EO was added to a 5 mL flask and volumetrically diluted with high-performance liquid chromatography (HPLC)-grade hexane. A 1 µL volume of the diluted sample was injected into the system. Before analysis, the samples were filtered using a hydrophilic Millipore needle microfilter (PTFE, Luer; Merck KGaA, Darmstadt, Germany) with dimensions of 13 mm/25 mm and a membrane pore size of 0.22/0.45/1.2 µm. The GC/MS system was equipped with a split/splitless injector and an AOC-20i autosampler were used for the analysis. The capillary column characteristics included fused silica (Thermo Fisher Scientific, Waltham, MA, USA) with a length of 30 m × 0.32 mm I.D. × 0.5 µm. The oven temperature was programmed at 50 °C for 4 min, followed by a 10 °C/min increase up to 220 °C, and the latter temperature was maintained for 2 min. The carrier gas was helium (99.99%) with a mobile phase flow rate of 1.10 mL/min, a linear velocity of 40 cm/s, a purge flow of 3.00 mL/min, and a split ratio of 25:1. The mass spectra of the detected compounds were compared with those available in the FFNSC4, NIST, and Wiley mass spectral libraries installed in the GC/MS LabSolutions software, version 5.11 using a spectral similarity threshold of 80% or higher. Since compound assignment was based on mass spectral library matching without confirmation by authentic standards or retention indices, the compounds were considered tentatively identified.

### 4.4. Antifungal Screening

The microorganism *C. albicans* (ATCC 10231) was used to determine the antifungal activity of the EO from *M. quixos* leaves. The strain was purchased from Medibac Laboratories in Guayaquil, Ecuador, and stored at −80 °C in the microbiology laboratory of the Universidad Estatal Amazónica until needed.

The *C. albicans* strain was reactivated by incubation in sealed test tubes containing potato dextrose agar (PDA; BD Bioxon^®^, Becton Dickinson, Cuautitlán Izcalli, Mexico) at a concentration of 39 g/L in water. Incubation was carried out at 30 °C for 48 h in accordance with the procedure described by Feldman et al. [61]. Then, the strain was diluted in Sabouraud dextrose broth at a concentration of 30 g/L until a suspension with a turbidity equivalent to 0.5 on the McFarland scale was obtained. The absorbance of the McFarland standard and the microbial suspension was determined at 625 nm using a Lambda 25 UV/VIS spectrophotometer (PerkinElmer, Waltham, MA, USA). The measurement process continued until the turbidity of the microbial suspension was equivalent to the 0.5 McFarland standard.

### 4.5. Determination of the Inhibitory Effect of M. quixos EO

The antifungal properties of *M. quixos* EO against *C. albicans* were assessed using the Kirby–Bauer disk diffusion method [62]. Initially, 1000 µL of the *C. albicans* suspension adjusted to 0.5 McFarland was inoculated onto each PDA plate and evenly spread over the agar surface to ensure uniform microbial growth. Subsequently, sterile 5 mm filter paper disks were placed on the inoculated agar surface. The EO was emulsified in Tween 20 solution (2% *v*/*v*) and prepared according to the concentrations established in the experimental design (see Table 2). A volume of 20 µL of each EO dilution was applied to the corresponding disk. Commercial ketoconazole 2% (*w*/*v*) was used directly as the positive antifungal control without further dilution. The plates were incubated at 30 °C for 48 h. After incubation, antifungal activity was determined by measuring the diameter of the inhibition zone formed around each disk.

### 4.6. Experiment Design and Model Fitting

A one-factor quadratic polynomial modeling approach was used to evaluate the relationship between *M. quixos* EO concentration and the inhibition zone diameter. The experimental matrix was generated using the Optimal (Custom) design option in Design-Expert software, version 13.0.5.0 (Stat-Ease Inc., Minneapolis, MN, USA) and included 15 experimental runs within the concentration range of 20–500 µL/mL. The design included model points (3), replicate points (5), lack-of-fit points (5), and additional center points (2). The inhibition zone diameter was used as the response variable. Model fitting was conducted using a second-order quadratic equation, expressed as follows:

Y = β_0_ + β_1_ X + β_2_ X^2^ + ε (2)

where Y represents the inhibition halo diameter (mm), X the concentration of the essential oil (μL/mL), β_0_ the independent term, β_1_ and β_2_ the linear and quadratic regression coefficients, respectively, and ε the error term. An ANOVA was performed to assess the statistical significance of the independent variable and its quadratic effect, considering a significance level of *p* < 0.05, ensuring the adequacy of the model in representing the experimental data.

### 4.7. Computational Target Fishing and Homology Mapping

To identify potential molecular targets of the EO compounds against *C. albicans*, a computational target fishing approach was implemented. Initial target prediction was performed using the SEA platform [63,64], which predicts protein targets based on the chemical similarity of ligands to known bioactive compounds. Because predicted targets may correspond to different organisms, a homology mapping procedure was performed to identify the corresponding fungal orthologs in *C. albicans* (taxid: 5476). Predicted targets were mapped to RefSeq Protein sequence codes using the UniProt database. Subsequently, protein–protein homology searches were conducted using the NCBI BLAST+ v2.17.0 server, with *C. albicans* as the reference organism. Homologous proteins were retained when the BLAST alignment covered at least 70% of the query sequence and showed at least 35% sequence identity. These validated fungal targets were then considered for molecular docking analysis to explore the possible binding mechanisms of *M. quixos* EO constituents.

### 4.8. Molecular Docking Study

The molecular docking study was conducted to investigate the interactions between the bioactive compounds identified in the essential oil of *M. quixos* and specific target proteins of *C. albicans* and Candida tropicalis using the study by Benhniya et al. [52] as a reference. The selected Protein Data Bank (PDB) structures were chosen based on target relevance, crystallographic quality, and the availability of fungal protein structures suitable for docking. The three-dimensional crystallographic structures of the target enzymes were downloaded from the Protein Data Bank (https://www.rcsb.org; accessed on 17 October 2025) in .pdb format. The 14-α-demethylase (CYP51) (PDB ID: 5TZ1; resolution: 2.00 Å) [52], corresponds to *C. albicans* and was selected because of its central role in ergosterol biosynthesis and its relevance as an antifungal target. The *exo*-*β*-(1,3)-glucanase (PDB ID: 1EQC; resolution: 1.85 Å) also corresponds to *C. albicans* and was selected because of its involvement in fungal cell wall remodeling [65]. The Δ(14)-sterol reductase (PDB ID: 4QUV; resolution: 2.74 Å) [66], corresponds to *S. cerevisiae* and was used as a homologous fungal surrogate for the corresponding *C. albicans* target, supported by the homology mapping procedure described above.

Protein preparation was performed using UCSF Chimera v1.19 (University of California, San Francisco, CA, USA) [67]. All non-essential components, such as water molecules, exogenous ligands, and irrelevant ions, were removed. The coordinates of the active site for each protein were identified based on the position of the co-crystallized ligand present in the original structures. Subsequently, AutoDockTools v1.5.7 (The Scripps Research Institute, La Jolla, CA, USA) [67] was used to complete receptor preparation. Polar hydrogens and Kollman partial charges were added, and the structures were saved in .pdbqt format, compatible with the AutoDock Vina calculation engine [68].

The 22 compounds identified in the essential oil of *M. quixos* using GC/MS were prepared as ligands, together with ketoconazole, which was used as the reference compound. The compound structures were obtained from PubChem (https://pubchem.ncbi.nlm.nih.gov/, accessed on 25 October 2025) and optimized using the MMFF94 force field in Avogadro v1.2.0 (Open Chemistry Project, Pittsburgh, PA, USA). The optimized molecules were saved in .mol2 format [69]. Subsequently, the files were processed in AutoDockTools v1.5.7, where polar hydrogens were added, Gasteiger charges were assigned, and rotatable carbons were defined. Finally, the ligands were saved in .pdbqt format for subsequent molecular docking analysis. No explicit pH- or temperature-dependent docking simulations were performed. Therefore, the docking results correspond to the ligand and receptor protonation states generated during the standard AutoDockTools preparation workflow under standard AutoDock Vina conditions.

To validate the methodology, the molecular redocking approach was applied to the three target proteins using their native ligands: castanospermine (CTS; 1EQC), dihydro-nicotinamide-adenine-dinucleotide phosphate (NADPH) (NDP; 4QUV), and protoporphyrin IX containing Fe (HEM; 5TZ1). The same preparation procedure and calculation parameters described above were applied to all ligands. A validation criterion of root mean square deviation (RMSD) of <3 Å was used, calculated using VMD v2.0 (University of Illinois at Urbana–Champaign, IL, USA) [69]. See Supplementary Materials S2.

Finally, computational modeling studies were carried out to generate the binding complex and predict the binding affinities between the natural compounds and selected enzymatic receptors. Rigid-receptor molecular docking calculations were performed for each system using AutoDock Vina v1.2.0 (The Scripps Research Institute, La Jolla, CA, USA) [68]. The coordinates and dimensions of the grid box were carefully defined around the known catalytic cavities to encompass the entire binding pocket, allowing the ligands unrestricted exploration of the active site. The grid box center coordinates, box dimensions, grid spacing, exhaustiveness value, and number of docking modes used for each target protein are summarized in Table 6.

The selection of the best-docked conformations was fundamentally based on the most negative binding energy scores (expressed in kcal/mol) and the physical feasibility of the intermolecular contacts. Finally, to thoroughly analyze the structural basis of the binding affinity, spatial interaction diagrams were generated using UCSF Chimera v1.19 [67] and LigPlot+ [70] to map the key interacting residues, hydrophobic contacts, and structural stabilization mechanisms within the binding pockets.

## 5. Conclusions

*M. quixos* essential oil showed relevant in vitro antifungal activity against *C. albicans*, reaching an inhibitory capacity of 87.3% relative to ketoconazole under the same assay conditions. Chemical characterization revealed a phenylpropanoid-rich profile dominated by (*E*)-cinnamaldehyde, accompanied by monoterpenes and sesquiterpenes. The one-factor quadratic model adequately described the relationship between EO concentration and inhibition halo diameter, showing high goodness of fit and predictive capacity within the evaluated concentration range. Molecular docking provided complementary in silico evidence suggesting that, although (*E*)-cinnamaldehyde was the major constituent, several sesquiterpenes, particularly *α*-copaene, *α*-guaiene, and spathulenol, may also contribute to the antifungal response through favorable predicted interactions with fungal targets involved in ergosterol biosynthesis and cell wall remodeling. Overall, these findings support the potential of *M. quixos* EO as a source of anticandidal compounds, while confirming that the results should be interpreted as preliminary in vitro and in silico evidence. Further studies involving additional fungal strains, toxicity and selectivity assays, in vivo models, and complementary computational analyses are required to validate its efficacy, safety, and mechanism of action.

## Figures and Tables

**Table 1:** Chemical Composition of the EO Obtained from *M. quixos*.

Peak	Retention Time	Area (%)	Name	Chemical Class
1	5.977	9.63	*α*-Pinene	Monoterpene
2	6.515	7.08	*β*-Pinene	Monoterpene
3	7.162	1.36	*β*-Terpinyl acetate	Monoterpene ester
4	8.447	0.81	Benzenepropanal	Aromatic aldehyde
5	9.094	0.31	*α*-Terpineol	Monoterpene alcohol
6	9.237	0.61	(*Z*)-Cinnamaldehyde	Aromatic aldehyde
7	9.995	47.20	(*E*)-Cinnamaldehyde	Aromatic aldehyde
8	11.82	0.92	Elixene	Sesquiterpene
9	12.05	4.63	Methyl cinnamate	Aromatic ester
10	12.63	1.93	*α*-Copaene	Sesquiterpene
11	12.84	0.64	*α*-Guaiene	Sesquiterpene
12	13.19	1.16	(*E*)-Cinnamyl acetate	Aromatic ester
13	13.49	10.80	Caryophyllene	Sesquiterpene
14	14.15	5.37	*α*-Humulene	Sesquiterpene
15	14.26	0.58	*cis*-Isomethyleugenol	Phenylpropanoid
16	15.00	1.33	Bicyclogermacrene	Sesquiterpene
17	15.30	0.31	*β*-Copaene	Sesquiterpene
18	15.46	0.74	*δ*-Cadinene	Sesquiterpene
19	16.39	0.31	*cis*-Caryophyllene	Sesquiterpene
20	16.54	0.57	Spathulenol	Sesquiterpenic alcohol
21	16.69	2.98	14-Hydroxycaryophyllene	Sesquiterpenic alcohol
22	17.21	0.77	Humulene epoxide II	Sesquiterpenic ether

**Table 2:** Experimental and predicted inhibition halo values for the antifungal activity of *M. quixos* EO against *C. albicans*.

Run	Concentration (µL/mL)	Inhibition Halo (mm)	Predicted Value (mm)
1	260	12.8	12.4
2	340	13.9	14.0
3	260	12.5	12.4
4	500	16.0	16.1
5	180	9.60	10.4
6	500	15.0	16.1
7	20	5.00	5.34
8	260	13.0	12.4
9	500	17.0	16.1
10	20	6.00	5.34
11	20	5.50	5.34
12	260	12.7	12.4
13	260	12.5	12.4
14	140	8.50	9.30
15	380	14.6	14.7

**Table 3:** ANOVA results for the quadratic model fitted to the antifungal activity of *M. quixos* EO against *C. albicans*.

Source	Sum of Squares	df	Mean Square	F-Value	*p*-Value
Model	201	2	101	266	<0.0001
A-Conc	192	1	193	509	<0.0001
A^2^	9.16	1	9.16	24.2	0.0004
Residual	4.54	12	0.378		
Lack of Fit	1.86	4	0.465	1.39	0.320
Pure Error	2.68	8	0.335		
Cor Total	206	14			

**Table 4:** Selected molecular targets identified through target fishing for *M. quixos* essential oil constituents.

UniProKB Entry	ID ^(a)^	Description	Structure Source ^(b)^	Identification	PDB Template
P10613	LDM	Lanosterol 14-*α*- demethylase (LDM)	PDB	SEA/SwissTargetPrediction	5TZ1
P29717	EBG	Glucan 1,3-*β*-glucosidase (EC 2.4.1.)	PDB	SEA/SwissTargetPrediction	1EQC
A0A1D8PIC7	DSR	Δ(14)-sterol reductase ERG24	PDB	SEA/SwissTargetPrediction	4QUV
A0A1D8PDA0	SRP	Sepiapterin reductase family protein	AlphaFold/Swiss-Model	SEA/SwissTargetPrediction	6UHX
A0A1D8PPY3	AMI	Amidase	AlphaFold/Swiss-Model	SEA/SwissTargetPrediction	6KVR
Q92206	SQE	Squalene epoxidase ERG1 (SE) (EC 1.14.14.17)	AlphaFold/Swiss-Model	SEA/SwissTargetPrediction	6C6N
Q5A399	PHO	Negative regulator of the PHO system (EC 2.7.11.22) (Serine/threonine-protein kinase PHO85)	AlphaFold/Swiss-Model	SEA/SwissTargetPrediction	8WX7
A0A1D8PDA6	G6P	Glucose-6-phosphate 1-dehydrogenase (EC 1.1.1.49)	AlphaFold/Swiss-Model	SEA/SwissTargetPrediction	4KRD
A0A1D8PEG2	MAP	Mitogen-activated protein kinase	AlphaFold/Swiss-Model	SEA/SwissTargetPrediction	6E07

^(a)^ The ID of each target throughout the manuscript. ^(b)^ Source of the structural model: Protein Data Bank (PDB), homology model (Swiss-Model), or AlphaFold repository (AlphaFold). Note: SEA = Similarity Ensemble Approach; PDB = Protein Data Bank.

**Table 5:** Molecular docking affinities of the compounds identified in the essential oil of *M. quixos* and ketoconazole against 14-*α*-demethylase, Δ(14)-sterol reductase, and exo-*β*-(1,3)-glucanase.

	Protein		
	14-*α*-Demethylase	Δ(14)-Sterol Reductase	Exo-*β*-(1,3)-Glucanase
Molecule	Affinity (kcal/mol)		
*α*-Pinene	−6.0	−5.9	−6.3
*β*-Pinene	−5.8	−5.7	−6.3
*β*-Terpinyl acetate	−6.8	−5.9	−7.5
Benzenepropanal	−5.9	−6.2	−5.7
*α*-Terpineol	−6.6	−6.2	−6.5
(*Z*)-Cinnamaldehyde	−6.0	−6.2	−6.4
(*E*)-Cinnamaldehyde	−6.2	−6.0	−6.0
Elixene	−7.0	−6.0	−7.7
Methyl cinnamate	−6.7	−6.3	−6.5
*α*-Copaene	−7.9	−7.1	−8.1
*α*-Guaiene	−7.3	−6.8	−8.2
(*E*)-Cinnamyl acetate	−7.2	−6.3	−7.0
Caryophyllene	−7.1	−6.8	−8.0
*α*-Humulene	−7.2	−6.4	−7.7
*cis*-Isomethyleugenol	−6.3	−6.2	−6.4
Bicyclogermacrene	−6.9	−6.5	−7.0
*β*-Copaene	−7.5	−6.7	−8.0
*δ*-Cadinene	−7.0	−6.8	−7.7
*cis*-Caryophyllene	−7.4	−6.5	−7.9
Spathulenol	−6.9	−6.9	−8.2
14-Hydroxycaryophyllene	−7.2	−6.6	−8.0
Humulene epoxide II	−7.1	−6.5	−8.1
Ketoconazole	−10.8	−9.5	−9.2

**Table 6:** Grid box parameters used for molecular docking calculations.

Target Protein	PDB ID	Center X	Center Y	Center Z	Box Size X × Y × Z (Å)	Grid Spacing (Å)	Exhaustiveness	Docking Modes
Exo-*β*-(1,3)-glucanase	1EQC	34.75	36.65	56.21	20 × 20 × 20	0.375	16	9
Δ(14)-sterol reductase	4QUV	−21.30	−9.16	26.43	30 × 30 × 30	0.375	16	9
14-*α*-demethylase	5TZ1	64.16	71.32	2.71	34 × 30 × 32	0.375	16	9

## Data Availability

Additional data supporting the findings of this study are available from the corresponding author upon reasonable request.

## Supplementary Materials

The following supporting information can be downloaded at: https://www.mdpi.com/article/10.3390/molecules31111891/s1, S1: Target fishing and homology mapping for molecular target selection. Table S1: Selected molecular targets identified through target fishing for *M. quixos* essential oil constituents. Table S2: Consolidated list of unique *C. albicans* homologous candidates identified through target fishing and homology mapping. Table S3: Complete RefSeq target-identification output used for homology mapping against *C. albicans*. S2: Validation of the molecular docking protocol by redocking the native ligands of 1EQC, 4QUV, and 5TZ1. Figure S1: Redocking validation of castanospermine (CTS) in *exo*-*β*-(1,3)-glucanase (PDB ID: 1EQC). Figure S2: Redocking validation of NADPH dihydro-nicotinamide-adenine-dinucleotide phosphate (NDP) in Δ(14)-sterol reductase (PDB ID: 4QUV). Figure S3: Redocking validation of protoporphyrin IX containing Fe (HEM) in 14-*α*-demethylase (PDB ID: 5TZ1).
