# Supplementary material for: Quadratic Concentration–Response Modeling and Molecular Docking of Mespilodaphne quixos (Lam.) Rohwer Essential Oil Against Candida albicans
Source: Molecules. 2026 Jun 1;31(11):1891. doi: 10.3390/molecules31111891 (PMC13257838; doi:10.3390/molecules31111891)
Supplement: Supplementary file 1 [file molecules-31-01891-s001.zip › Supplementary_material S1.pdf]

# Quadratic Concentration-Response Modeling and Molecular Docking of *Mespilodaphne quixos* (Lam.) Rohwer Essential Oil Against *Candida albicans*

Yasiel Arteaga-Crespo, Yudel García-Quintana, Yendrek Velásquez-López, Matteo Radice, Mariana Magdalena Conforme-García, Jannys Lizeth Rivera-Barreto, José Blanco-Salas, Reinier Abreu-Naranjo*

* Correspondence: rabreu@uea.edu.ec

**Supplementary Material S1: Target fishing and homology mapping for molecular target selection. This single supplementary document contains Tables S1–S3.**

This supplementary material provides the target-fishing and homology-mapping outputs used to support the selection of fungal protein targets for molecular docking. Candidate proteins were obtained from ligand-based target prediction and subsequently mapped to *C. albicans* homologues. Selected molecular targets were prioritized based on biological relevance, availability of suitable structural templates, and involvement in ergosterol biosynthesis or cell-wall-related processes.

**Table S1. Prioritized molecular targets identified through target fishing and homology mapping for *M. quixos* EO constituents.**

| UniProKB entry | ID(a) | Description | Structure source(b) | Identification | PDB template |
|---|---|---|---|---|---|
| P10613 | LDM | Lanosterol 14-α- demethylase (LDM) | PDB | SEA/SwissTargetPrediction | 5TZ1 |
| P29717 | EBG | Glucan 1,3-β-glucosidase (EC 2.4.1.) | PDB | SEA/SwissTargetPrediction | 1EQC |
| A0A1D8PIC7 | DSR | Δ(14)-sterol reductase ERG24 | PDB | SEA/SwissTargetPrediction | 4QUV |
| A0A1D8PDA0 | SRP | Sepiapterin reductase family protein | Alphafold/Swiss-Model | SEA/SwissTargetPrediction | 6UHX |
| A0A1D8PPY3 | AMI | Amidase | Alphafold/Swiss-Model | SEA/SwissTargetPrediction | 6KVR |
| Q92206 | SQE | Squalene epoxidase ERG1 (SE) (EC 1.14.14.17) | Alphafold/Swiss-Model | SEA/SwissTargetPrediction | 6C6N |
| Q5A399 | PHO | Negative regulator of the PHO system (EC 2.7.11.22) (Serine/threonine-protein kinase PHO85) | Alphafold/Swiss-Model | SEA/SwissTargetPrediction | 8WX7 |
| A0A1D8PDA6 | G6P | Glucose-6-phosphate 1-dehydrogenase (EC 1.1.1.49) | Alphafold/Swiss-Model | SEA/SwissTargetPrediction | 4KRD |
| A0A1D8PEG2 | MAP | Mitogen-activated protein kinase | Alphafold/Swiss-Model | SEA/SwissTargetPrediction | 6E07 |

(a) ID used for each target in the manuscript. (b) Source of the structural model: Protein Data Bank (PDB), homology model (Swiss-Model), or AlphaFold repository.

**Table S2. Unique *C. albicans* homologous target candidates obtained from the uploaded ligand-specific RefSeq target-identification files.**

| RefSeq accession | UniProtKB entry | Entry name | Protein name | Gene names | Organism | Length | Source dataset(s) |
|---|---|---|---|---|---|---|---|
| XP_019330641.1 | A0A1D8PDA0 | A0A1D8PDA0_CANAL | Sepiapterin reductase family protein | CAALFM_C104430CA orf19.5194.1 | Candida albicans (strain SC5314 / ATCC MYA-2876) (Yeast) | 253 | β-copaene |
| XP_019330642.1 | A0A1D8PDA4 | A0A1D8PDA4_CANAL | Fma1p | FMA1 CAALFM_C104450CA orf19.12660 | Candida albicans (strain SC5314 / ATCC MYA-2876) (Yeast) | 253 | β-copaene |
| XP_019330643.1 | A0A1D8PDA6 | A0A1D8PDA6_CANAL | Negative regulator of the PHO system (EC 2.7.11.22) (Serine/threonine-protein kinase PHO85) | PHO85 CAALFM_C104520CA orf19.14136 | Candida albicans (strain SC5314 / ATCC MYA-2876) (Yeast) | 332 | α-copaene |
| XP_718826.2 | A0A1D8PDV5 | A0A1D8PDV5_CANAL | Rho family GTPase | RAC1 CAALFM_C106730WA orf19.13617 | Candida albicans (strain SC5314 / ATCC MYA-2876) (Yeast) | 236 | spathulenol |
| XP_019330676.1 | A0A1D8PE91 | A0A1D8PE91_CANAL | Tyrosine protein phosphatase | LTP1 CAALFM_C108260CA orf19.12570 | Candida albicans (strain SC5314 / ATCC MYA-2876) (Yeast) | 168 | α-copaene |

| | | | | | | | |
|---|---|---|---|---|---|---|---|
| XP_723440.2 | A0A1D8PEG2 | A0A1D8PEG2_CANAL | Glucose-6-phosphate 1-dehydrogenase (EC 1.1.1.49) | ZWF1 CAALFM_C108980CA orf19.12218 | Candida albicans (strain SC5314 / ATCC MYA-2876) (Yeast) | 507 | α-copaene |
| XP_019330730.1 | A0A1D8PFT5 | A0A1D8PFT5_CANAL | Mitogen-activated protein kinase | CSK1 CAALFM_C114010WA orf19.7208 | Candida albicans (strain SC5314 / ATCC MYA-2876) (Yeast) | 374 | α-copaene |
| XP_019330773.1 | A0A1D8PH96 | A0A1D8PH96_CANAL | GTP-binding protein RHO3 | RHO3 CAALFM_C205030CA orf19.11018 | Candida albicans (strain SC5314 / ATCC MYA-2876) (Yeast) | 210 | spathulenol |
| XP_714866.2 | A0A1D8PHU1 | A0A1D8PHU1_CANAL | cAMP-dependent protein kinase (EC 2.7.11.11) | TPK2 CAALFM_C207210CA orf19.9817 | Candida albicans (strain SC5314 / ATCC MYA-2876) (Yeast) | 413 | spathulenol |
| XP_714828.2 | A0A1D8PHV3 | A0A1D8PHV3_CANAL | glucan 1,3-beta-glucosidase (EC 3.2.1.58) | SPR1 CAALFM_C206840WA orf19.9779 | Candida albicans (strain SC5314 / ATCC MYA-2876) (Yeast) | 525 | spathulenol |
| XP_019330798.1 | A0A1D8PHZ5 | A0A1D8PHZ5_CANAL | Rho family GTPase | RHO2 CAALFM_C207750WA orf19.2204.2 | Candida albicans (strain SC5314 / ATCC MYA-2876) (Yeast) | 187 | spathulenol |
| XP_716013.2 | A0A1D8PI24 | A0A1D8PI24_CANAL | D-arabinose 1-dehydrogenase (NAD(P)(+)) | ARA1 CAALFM_C208130WA orf19.9718 | Candida albicans (strain SC5314 / ATCC MYA-2876) (Yeast) | 326 | α-copaene, spathulenol |
| XP_714460.2 | A0A1D8PI71 | ERG9_CANAL | Squalene synthase ERG9 (SQS) (SS) (EC 2.5.1.21) (Ergosterol biosynthesis protein 9) (FPP:FPP farnesyltransferase ERG9) (Farnesyl-diphosphate farnesyltransferase ERG9) | ERG9 orf19.3616 CAALFM_C208610WA | Candida albicans (strain SC5314 / ATCC MYA-2876) (Yeast) | 448 | α-guaiene |
| XP_710679.2 | A0A1D8PIC7 | ERG24_CANAL | Delta(14)-sterol reductase ERG24 (EC 1.3.1.70) (C-14 sterol reductase ERG24) (Sterol C14-reductase ERG24) | ERG24 orf19.1598 CAALFM_C209400CA | Candida albicans (strain SC5314 / ATCC MYA-2876) (Yeast) | 448 | α-copaene, spathulenol |
| XP_715530.2 | A0A1D8PMM1 | A0A1D8PMM1_CANAL | DNA topoisomerase 2 (EC 5.6.2.2) | TOP2 CAALFM_C406600WA orf19.10391 | Candida albicans (strain SC5314 / ATCC MYA-2876) (Yeast) | 1461 | α-copaene |
| XP_720624.2 | A0A1D8PNK3 | A0A1D8PNK3_CANAL | D-xylose reductase [NAD(P)H] (EC 1.1.1.307) | GRE3 CAALFM_C502930CA orf19.11792 | Candida albicans (strain SC5314 / ATCC MYA-2876) (Yeast) | 371 | α-copaene, spathulenol |
| XP_719100.2 | A0A1D8PPY3 | A0A1D8PPY3_CANAL | Amidase | CAALFM_C602660CA orf19.12982 | Candida albicans (strain SC5314 / ATCC MYA-2876) (Yeast) | 572 | β-copaene |
| XP_716991.2 | A0A1D8PR62 | A0A1D8PR62_CANAL | amidase (EC 3.5.1.4) | CAALFM_C702920WA orf19.12636 | Candida albicans (strain SC5314 / ATCC MYA-2876) (Yeast) | 573 | β-copaene |
| XP_715894.2 | A0A1D8PT38 | A0A1D8PT38_CANAL | Mitogen-activated protein kinase (EC 2.7.11.24) | CEK2 CAALFM_CR05940WA orf19.8091 | Candida albicans (strain SC5314 / ATCC MYA-2876) (Yeast) | 372 | α-copaene, spathulenol |
| XP_715774.1 | O42825 | RHO1_CANAL | GTP-binding protein RHO1 | RHO1 CAALFM_CR02860WA CaO19.10362 CaO19.2843 | Candida albicans (strain SC5314 / ATCC MYA-2876) (Yeast) | 198 | spathulenol |
| XP_711878.1 | P0CY33 | CDC42_CANAL | Cell division control protein 42 homolog | CDC42 CAALFM_C108450CA CaO19.390 CaO19.8020 | Candida albicans (strain SC5314 / ATCC MYA-2876) (Yeast) | 191 | spathulenol |
| XP_716761.1 | P10613 | CP51_CANAL | Lanosterol 14-alpha demethylase (LDM) (EC 1.14.14.154) (Cytochrome P450 51) (Cytochrome P450-14DM) (Cytochrome P450-LIA1) (CYPLI) (Ergosterol biosynthesis protein 11) (Sterol 14-alpha demethylase) | ERG11 CYP51 ERG16 CAALFM_C500660CA CaO19.922 | Candida albicans (strain SC5314 / ATCC MYA-2876) (Yeast) | 528 | α-copaene, spathulenol, α-guaiene, β-copaene |
| XP_721488.2 | P29717 | EXG1_CANAL | Glucan 1,3-beta-glucosidase (EC 2.4.1.-) (EC 3.2.1.58) (Exo-1,3-beta-glucanase) | XOG1 EXG EXG1 XOG CAALFM_C102990CA Ca49C10.05 CaO19.10507 CaO19.2990 | Candida albicans (strain SC5314 / ATCC MYA-2876) (Yeast) | 438 | α-copaene, spathulenol, α-guaiene |
| XP_713486.1 | P43063 | CDK1_CANAL | Cyclin-dependent kinase 1 (CDK1) (EC 2.7.11.22) (Cell division control protein 28) (Cell division protein kinase 2) | CDC28 CDK1 CAALFM_CR06050WA CaO19.11337 CaO19.3856 | Candida albicans (strain SC5314 / ATCC MYA-2876) (Yeast) | 317 | α-copaene, spathulenol |
| XP_722612.2 | Q04782 | ERG7_CANAL | Lanosterol synthase (EC 5.4.99.7) (2,3-epoxysqualene--lanosterol cyclase) (Ergosterol biosynthesis protein 7) (Oxidosqualene--lanosterol cyclase) (OSC) | ERG7 CAALFM_C202460WA CaO19.1570 | Candida albicans (strain SC5314 / ATCC MYA-2876) (Yeast) | 728 | α-copaene, α-guaiene |
| XP_710404.1 | Q59KZ2 | Q59KZ2_CANAL | Peptidyl-prolyl cis-trans isomerase (EC 5.2.1.8) | ESS1 CAALFM_C104410CA orf19.12663 | Candida albicans (strain SC5314 / ATCC MYA-2876) (Yeast) | 177 | α-guaiene |
| XP_713148.1 | Q59U59 | Q59U59_CANAL | Aspartate protease | APR1 CAALFM_C207400CA orf19.9447 | Candida albicans (strain SC5314 / ATCC MYA-2876) (Yeast) | 419 | α-copaene, α-guaiene |
| XP_715542.2 | Q5A1D3 | ERK1_CANAL | Extracellular signal-regulated kinase 1 (ERK1) (EC 2.7.11.24) (MAP kinase 1) (MAPK 1) | CEK1 ERK1 CAALFM_C406480CA CaO19.10404 CaO19.2886 | Candida albicans (strain SC5314 / ATCC MYA-2876) (Yeast) | 421 | α-copaene, spathulenol |

| | | | | | | | |
|---|---|---|---|---|---|---|---|
| XP_716206.1 | Q5A399 | Q5A399_CANAL | protein-tyrosine-phosphatase (EC 3.1.3.48) | PTP1 CAALFM_CR08070WA orf19.13722 | Candida albicans (strain SC5314 / ATCC MYA-2876) (Yeast) | 372 | β-copaene |
| XP_718680.1 | Q5AAG6 | MKC1_CANAL | Mitogen-activated protein kinase MKC1 (MAP kinase MKC1) (EC 2.7.11.24) | MKC1 CAALFM_CR00120CA CaO19.7523 | Candida albicans (strain SC5314 / ATCC MYA-2876) (Yeast) | 509 | spathulenol |
| XP_719855.1 | Q5ADT3 | Q5ADT3_CANAL | NADP-dependent oxidoreductase domain-containing protein | CAALFM_C307330WA orf19.14050 | Candida albicans (strain SC5314 / ATCC MYA-2876) (Yeast) | 289 | α-copaene, spathulenol |
| XP_719854.1 | Q5ADT4 | Q5ADT4_CANAL | Glycerol 2-dehydrogenase (NADP(+)) | GCY1 CAALFM_C307340WA orf19.14049 | Candida albicans (strain SC5314 / ATCC MYA-2876) (Yeast) | 295 | α-copaene, spathulenol |
| XP_721393.1 | Q5AGY1 | Q5AGY1_CANAL | amidase (EC 3.5.1.4) | CAALFM_C701670WA orf19.13910 | Candida albicans (strain SC5314 / ATCC MYA-2876) (Yeast) | 579 | β-copaene |
| XP_720961.1 | Q5AHF9 | Q5AHF9_CANAL | Glucosamine 6-phosphate N-acetyltransferase (EC 2.3.1.4) | GNA1 CAALFM_C203870WA | Candida albicans (strain SC5314 / ATCC MYA-2876) (Yeast) | 149 | α-copaene |
| XP_721535.1 | Q5AI15 | PABP_CANAL | Polyadenylate-binding protein, cytoplasmic and nuclear (PABP) (Poly(A)-binding protein) (Polyadenylate tail-binding protein) | PAB1 CAALFM_C103370WA CaO19.10555 CaO19.3037 | Candida albicans (strain SC5314 / ATCC MYA-2876) (Yeast) | 629 | spathulenol |
| XP_721451.2 | Q5AIA1 | EXG2_CANAL | Glucan 1,3-beta-glucosidase 2 (EC 3.2.1.58) (Exo-1,3-beta-glucanase 2) | EXG2 CAALFM_C102630CA CaO19.10469 CaO19.2952 | Candida albicans (strain SC5314 / ATCC MYA-2876) (Yeast) | 479 | α-copaene, spathulenol, α-guaiene |
| XP_723574.1 | Q5AP71 | Q5AP71_CANAL | cAMP-dependent protein kinase (EC 2.7.11.11) | TPK1 CAALFM_C110220CA orf19.12357 | Candida albicans (strain SC5314 / ATCC MYA-2876) (Yeast) | 405 | α-copaene, spathulenol |
| XP_711894.1 | Q92206 | ERG1_CANAL | Squalene epoxidase ERG1 (SE) (EC 1.14.14.17) (Ergosterol biosynthesis protein 1) (Squalene monooxygenase ERG1) | ERG1 CAALFM_C108590CA CaO19.406 CaO19.8036 | Candida albicans (strain SC5314 / ATCC MYA-2876) (Yeast) | 496 | α-copaene, α-guaiene |
| XP_721016.1 | Q92207 | HOG1_CANAL | Mitogen-activated protein kinase HOG1 (MAP kinase HOG1) (EC 2.7.11.24) | HOG1 CAALFM_C203330CA CaO19.8514 CaO19.895 | Candida albicans (strain SC5314 / ATCC MYA-2876) (Yeast) | 377 | α-copaene, spathulenol |

Note: "α-copaene" corresponds to the uploaded file "RefSeq - C Albicans target identification-1.xlsx", which did not include a compound name in the file name. The unique-target table consolidates duplicate entries detected across ligand-specific files. The complete row-level target-fishing output is provided in Table S3.

## Table S3. Complete row-level target-fishing output from the ligand-specific RefSeq target-identification files.

| Source dataset | RefSeq accession | UniProtKB entry | Entry name | Protein name | Gene names | Organism | Length |
|---|---|---|---|---|---|---|---|
| α-copaene | XP_019330643.1 | A0A1D8PDA6 | A0A1D8PDA6_CANAL | Negative regulator of the PHO system (EC 2.7.11.22) (Serine/threonine-protein kinase PHO85) | PHO85 CAALFM_C104520CA orf19.14136 | Candida albicans (strain SC5314 / ATCC MYA-2876) (Yeast) | 332 |
| α-copaene | XP_019330676.1 | A0A1D8PE91 | A0A1D8PE91_CANAL | Tyrosine protein phosphatase | LTP1 CAALFM_C108260CA orf19.12570 | Candida albicans (strain SC5314 / ATCC MYA-2876) (Yeast) | 168 |
| α-copaene | XP_723440.2 | A0A1D8PEG2 | A0A1D8PEG2_CANAL | Glucose-6-phosphate 1-dehydrogenase (EC 1.1.1.49) | ZWF1 CAALFM_C108980CA orf19.12218 | Candida albicans (strain SC5314 / ATCC MYA-2876) (Yeast) | 507 |
| α-copaene | XP_019330730.1 | A0A1D8PFT5 | A0A1D8PFT5_CANAL | Mitogen-activated protein kinase | CSK1 CAALFM_C114010WA orf19.7208 | Candida albicans (strain SC5314 / ATCC MYA-2876) (Yeast) | 374 |
| α-copaene | XP_716013.2 | A0A1D8PI24 | A0A1D8PI24_CANAL | D-arabinose 1-dehydrogenase (NAD(P)(+)) | ARA1 CAALFM_C208130WA orf19.9718 | Candida albicans (strain SC5314 / ATCC MYA-2876) (Yeast) | 326 |
| α-copaene | XP_710679.2 | A0A1D8PIC7 | ERG24_CANAL | Delta(14)-sterol reductase ERG24 (EC 1.3.1.70) (C-14 sterol reductase ERG24) (Sterol C14-reductase ERG24) | ERG24 orf19.1598 CAALFM_C209400CA | Candida albicans (strain SC5314 / ATCC MYA-2876) (Yeast) | 448 |
| α-copaene | XP_715530.2 | A0A1D8PMM1 | A0A1D8PMM1_CANAL | DNA topoisomerase 2 (EC 5.6.2.2) | TOP2 CAALFM_C406600WA orf19.10391 | Candida albicans (strain SC5314 / ATCC MYA-2876) (Yeast) | 1461 |
| α-copaene | XP_720624.2 | A0A1D8PNK3 | A0A1D8PNK3_CANAL | D-xylose reductase [NAD(P)H] (EC 1.1.1.307) | GRE3 CAALFM_C502930CA orf19.11792 | Candida albicans (strain SC5314 / ATCC MYA-2876) (Yeast) | 371 |
| α-copaene | XP_715894.2 | A0A1D8PT38 | A0A1D8PT38_CANAL | Mitogen-activated protein kinase (EC 2.7.11.24) | CEK2 CAALFM_CR05940WA orf19.8091 | Candida albicans (strain SC5314 / ATCC MYA-2876) (Yeast) | 372 |
| α-copaene | XP_716761.1 | P10613 | CP51_CANAL | Lanosterol 14-alpha demethylase (LDM) (EC 1.14.14.154) (Cytochrome P450 51) (Cytochrome P450-14DM) (Cytochrome P450-LIA1) (CYPLI) (Ergosterol biosynthesis protein 11) (Sterol 14-alpha demethylase) | ERG11 CYP51 ERG16 CAALFM_C500660CA CaO19.922 | Candida albicans (strain SC5314 / ATCC MYA-2876) (Yeast) | 528 |

| | | | | | | | |
|---|---|---|---|---|---|---|---|
| α-copaene | XP_721488.2 | P29717 | EXG1_CANAL | Glucan 1,3-beta-glucosidase (EC 2.4.1.-) (EC 3.2.1.58) (Exo-1,3-beta-glucanase) | XOG1 EXG EXG1 XOG CAALFM_C102990CA Ca49C10.05 CaO19.10507 CaO19.2990 | Candida albicans (strain SC5314 / ATCC MYA-2876) (Yeast) | 438 |
| α-copaene | XP_713486.1 | P43063 | CDK1_CANAL | Cyclin-dependent kinase 1 (CDK1) (EC 2.7.11.22) (Cell division control protein 28) (Cell division protein kinase 2) | CDC28 CDK1 CAALFM_CR06050WA CaO19.11337 CaO19.3856 | Candida albicans (strain SC5314 / ATCC MYA-2876) (Yeast) | 317 |
| α-copaene | XP_722612.2 | Q04782 | ERG7_CANAL | Lanosterol synthase (EC 5.4.99.7) (2,3-epoxysqualene--lanosterol cyclase) (Ergosterol biosynthesis protein 7) (Oxidosqualene--lanosterol cyclase) (OSC) | ERG7 CAALFM_C202460WA CaO19.1570 | Candida albicans (strain SC5314 / ATCC MYA-2876) (Yeast) | 728 |
| α-copaene | XP_713148.1 | Q59U59 | Q59U59_CANAL | Aspartate protease | APR1 CAALFM_C207400CA orf19.9447 | Candida albicans (strain SC5314 / ATCC MYA-2876) (Yeast) | 419 |
| α-copaene | XP_715542.2 | Q5A1D3 | ERK1_CANAL | Extracellular signal-regulated kinase 1 (ERK1) (EC 2.7.11.24) (MAP kinase 1) (MAPK 1) | CEK1 ERK1 CAALFM_C406480CA CaO19.10404 CaO19.2886 | Candida albicans (strain SC5314 / ATCC MYA-2876) (Yeast) | 421 |
| α-copaene | XP_719855.1 | Q5ADT3 | Q5ADT3_CANAL | NADP-dependent oxidoreductase domain-containing protein | CAALFM_C307330WA orf19.14050 | Candida albicans (strain SC5314 / ATCC MYA-2876) (Yeast) | 289 |
| α-copaene | XP_719854.1 | Q5ADT4 | Q5ADT4_CANAL | Glycerol 2-dehydrogenase (NADP(+)) | GCY1 CAALFM_C307340WA orf19.14049 | Candida albicans (strain SC5314 / ATCC MYA-2876) (Yeast) | 295 |
| α-copaene | XP_720961.1 | Q5AHF9 | Q5AHF9_CANAL | Glucosamine 6-phosphate N-acetyltransferase (EC 2.3.1.4) | GNA1 CAALFM_C203870WA | Candida albicans (strain SC5314 / ATCC MYA-2876) (Yeast) | 149 |
| α-copaene | XP_721451.2 | Q5AIA1 | EXG2_CANAL | Glucan 1,3-beta-glucosidase 2 (EC 3.2.1.58) (Exo-1,3-beta-glucanase 2) | EXG2 CAALFM_C102630CA CaO19.10469 CaO19.2952 | Candida albicans (strain SC5314 / ATCC MYA-2876) (Yeast) | 479 |
| α-copaene | XP_723574.1 | Q5AP71 | Q5AP71_CANAL | cAMP-dependent protein kinase (EC 2.7.11.11) | TPK1 CAALFM_C110220CA orf19.12357 | Candida albicans (strain SC5314 / ATCC MYA-2876) (Yeast) | 405 |
| α-copaene | XP_711894.1 | Q92206 | ERG1_CANAL | Squalene epoxidase ERG1 (SE) (EC 1.14.14.17) (Ergosterol biosynthesis protein 1) (Squalene monooxygenase ERG1) | ERG1 CAALFM_C108590CA CaO19.406 CaO19.8036 | Candida albicans (strain SC5314 / ATCC MYA-2876) (Yeast) | 496 |
| α-copaene | XP_721016.1 | Q92207 | HOG1_CANAL | Mitogen-activated protein kinase HOG1 (MAP kinase HOG1) (EC 2.7.11.24) | HOG1 CAALFM_C203330CA CaO19.8514 CaO19.895 | Candida albicans (strain SC5314 / ATCC MYA-2876) (Yeast) | 377 |
| α-guaiene | XP_714460.2 | A0A1D8PI71 | ERG9_CANAL | Squalene synthase ERG9 (SQS) (SS) (EC 2.5.1.21) (Ergosterol biosynthesis protein 9) (FPP:FPP farnesyltransferase ERG9) (Farnesyl-diphosphate farnesyltransferase ERG9) | ERG9 orf19.3616 CAALFM_C208610WA | Candida albicans (strain SC5314 / ATCC MYA-2876) (Yeast) | 448 |
| α-guaiene | XP_716761.1 | P10613 | CP51_CANAL | Lanosterol 14-alpha demethylase (LDM) (EC 1.14.14.154) (Cytochrome P450 51) (Cytochrome P450-14DM) (Cytochrome P450-LIA1) (CYPLI) (Ergosterol biosynthesis protein 11) (Sterol 14-alpha demethylase) | ERG11 CYP51 ERG16 CAALFM_C500660CA CaO19.922 | Candida albicans (strain SC5314 / ATCC MYA-2876) (Yeast) | 528 |
| α-guaiene | XP_721488.2 | P29717 | EXG1_CANAL | Glucan 1,3-beta-glucosidase (EC 2.4.1.-) (EC 3.2.1.58) (Exo-1,3-beta-glucanase) | XOG1 EXG EXG1 XOG CAALFM_C102990CA Ca49C10.05 CaO19.10507 CaO19.2990 | Candida albicans (strain SC5314 / ATCC MYA-2876) (Yeast) | 438 |
| α-guaiene | XP_722612.2 | Q04782 | ERG7_CANAL | Lanosterol synthase (EC 5.4.99.7) (2,3-epoxysqualene--lanosterol cyclase) (Ergosterol biosynthesis protein 7) (Oxidosqualene--lanosterol cyclase) (OSC) | ERG7 CAALFM_C202460WA CaO19.1570 | Candida albicans (strain SC5314 / ATCC MYA-2876) (Yeast) | 728 |
| α-guaiene | XP_710404.1 | Q59KZ2 | Q59KZ2_CANAL | Peptidyl-prolyl cis-trans isomerase (EC 5.2.1.8) | ESS1 CAALFM_C104410CA orf19.12663 | Candida albicans (strain SC5314 / ATCC MYA-2876) (Yeast) | 177 |
| α-guaiene | XP_713148.1 | Q59U59 | Q59U59_CANAL | Aspartate protease | APR1 CAALFM_C207400CA orf19.9447 | Candida albicans (strain SC5314 / ATCC MYA-2876) (Yeast) | 419 |
| α-guaiene | XP_721451.2 | Q5AIA1 | EXG2_CANAL | Glucan 1,3-beta-glucosidase 2 (EC 3.2.1.58) (Exo-1,3-beta-glucanase 2) | EXG2 CAALFM_C102630CA CaO19.10469 CaO19.2952 | Candida albicans (strain SC5314 / ATCC MYA-2876) (Yeast) | 479 |
| α-guaiene | XP_711894.1 | Q92206 | ERG1_CANAL | Squalene epoxidase ERG1 (SE) (EC 1.14.14.17) (Ergosterol biosynthesis protein 1) (Squalene monooxygenase ERG1) | ERG1 CAALFM_C108590CA CaO19.406 CaO19.8036 | Candida albicans (strain SC5314 / ATCC MYA-2876) (Yeast) | 496 |
| β-copaene | XP_019330641.1 | A0A1D8PDA0 | A0A1D8PDA0_CANAL | Sepiapterin reductase family protein | CAALFM_C104430CA orf19.5194.1 | Candida albicans (strain SC5314 / ATCC MYA-2876) (Yeast) | 253 |
| β-copaene | XP_019330642.1 | A0A1D8PDA4 | A0A1D8PDA4_CANAL | Fma1p | FMA1 CAALFM_C104450CA orf19.12660 | Candida albicans (strain SC5314 / ATCC MYA-2876) (Yeast) | 253 |

| | | | | | | | |
|---|---|---|---|---|---|---|---|
| β-copaene | XP_719100.2 | A0A1D8PPY3 | A0A1D8PPY3_CANAL | Amidase | CAALFM_C602660CA orf19.12982 | Candida albicans (strain SC5314 / ATCC MYA-2876) (Yeast) | 572 |
| β-copaene | XP_716991.2 | A0A1D8PR62 | A0A1D8PR62_CANAL | amidase (EC 3.5.1.4) | CAALFM_C702920WA orf19.12636 | Candida albicans (strain SC5314 / ATCC MYA-2876) (Yeast) | 573 |
| β-copaene | XP_716761.1 | P10613 | CP51_CANAL | Lanosterol 14-alpha demethylase (LDM) (EC 1.14.14.154) (Cytochrome P450 51) (Cytochrome P450-14DM) (Cytochrome P450-LIA1) (CYPLI) (Ergosterol biosynthesis protein 11) (Sterol 14-alpha demethylase) | ERG11 CYP51 ERG16 CAALFM_C500660CA CaO19.922 | Candida albicans (strain SC5314 / ATCC MYA-2876) (Yeast) | 528 |
| β-copaene | XP_716206.1 | Q5A399 | Q5A399_CANAL | protein-tyrosine-phosphatase (EC 3.1.3.48) | PTP1 CAALFM_CR08070WA orf19.13722 | Candida albicans (strain SC5314 / ATCC MYA-2876) (Yeast) | 372 |
| β-copaene | XP_721393.1 | Q5AGY1 | Q5AGY1_CANAL | amidase (EC 3.5.1.4) | CAALFM_C701670WA orf19.13910 | Candida albicans (strain SC5314 / ATCC MYA-2876) (Yeast) | 579 |
| spathulenol | XP_718826.2 | A0A1D8PDV5 | A0A1D8PDV5_CANAL | Rho family GTPase | RAC1 CAALFM_C106730WA orf19.13617 | Candida albicans (strain SC5314 / ATCC MYA-2876) (Yeast) | 236 |
| spathulenol | XP_019330773.1 | A0A1D8PH96 | A0A1D8PH96_CANAL | GTP-binding protein RHO3 | RHO3 CAALFM_C205030CA orf19.11018 | Candida albicans (strain SC5314 / ATCC MYA-2876) (Yeast) | 210 |
| spathulenol | XP_714866.2 | A0A1D8PHU1 | A0A1D8PHU1_CANAL | cAMP-dependent protein kinase (EC 2.7.11.11) | TPK2 CAALFM_C207210CA orf19.9817 | Candida albicans (strain SC5314 / ATCC MYA-2876) (Yeast) | 413 |
| spathulenol | XP_714828.2 | A0A1D8PHV3 | A0A1D8PHV3_CANAL | glucan 1,3-beta-glucosidase (EC 3.2.1.58) | SPR1 CAALFM_C206840WA orf19.9779 | Candida albicans (strain SC5314 / ATCC MYA-2876) (Yeast) | 525 |
| spathulenol | XP_019330798.1 | A0A1D8PHZ5 | A0A1D8PHZ5_CANAL | Rho family GTPase | RHO2 CAALFM_C207750WA orf19.2204.2 | Candida albicans (strain SC5314 / ATCC MYA-2876) (Yeast) | 187 |
| spathulenol | XP_716013.2 | A0A1D8PI24 | A0A1D8PI24_CANAL | D-arabinose 1-dehydrogenase (NAD(P)(+)) | ARA1 CAALFM_C208130WA orf19.9718 | Candida albicans (strain SC5314 / ATCC MYA-2876) (Yeast) | 326 |
| spathulenol | XP_710679.2 | A0A1D8PIC7 | ERG24_CANAL | Delta(14)-sterol reductase ERG24 (EC 1.3.1.70) (C-14 sterol reductase ERG24) (Sterol C14-reductase ERG24) | ERG24 orf19.1598 CAALFM_C209400CA | Candida albicans (strain SC5314 / ATCC MYA-2876) (Yeast) | 448 |
| spathulenol | XP_720624.2 | A0A1D8PNK3 | A0A1D8PNK3_CANAL | D-xylose reductase [NAD(P)H] (EC 1.1.1.307) | GRE3 CAALFM_C502930CA orf19.11792 | Candida albicans (strain SC5314 / ATCC MYA-2876) (Yeast) | 371 |
| spathulenol | XP_715894.2 | A0A1D8PT38 | A0A1D8PT38_CANAL | Mitogen-activated protein kinase (EC 2.7.11.24) | CEK2 CAALFM_CR05940WA orf19.8091 | Candida albicans (strain SC5314 / ATCC MYA-2876) (Yeast) | 372 |
| spathulenol | XP_715774.1 | O42825 | RHO1_CANAL | GTP-binding protein RHO1 | RHO1 CAALFM_CR02860WA CaO19.10362 CaO19.2843 | Candida albicans (strain SC5314 / ATCC MYA-2876) (Yeast) | 198 |
| spathulenol | XP_711878.1 | P0CY33 | CDC42_CANAL | Cell division control protein 42 homolog | CDC42 CAALFM_C108450CA CaO19.390 CaO19.8020 | Candida albicans (strain SC5314 / ATCC MYA-2876) (Yeast) | 191 |
| spathulenol | XP_716761.1 | P10613 | CP51_CANAL | Lanosterol 14-alpha demethylase (LDM) (EC 1.14.14.154) (Cytochrome P450 51) (Cytochrome P450-14DM) (Cytochrome P450-LIA1) (CYPLI) (Ergosterol biosynthesis protein 11) (Sterol 14-alpha demethylase) | ERG11 CYP51 ERG16 CAALFM_C500660CA CaO19.922 | Candida albicans (strain SC5314 / ATCC MYA-2876) (Yeast) | 528 |
| spathulenol | XP_721488.2 | P29717 | EXG1_CANAL | Glucan 1,3-beta-glucosidase (EC 2.4.1.-) (EC 3.2.1.58) (Exo-1,3-beta-glucanase) | XOG1 EXG EXG1 XOG CAALFM_C102990CA Ca49C10.05 CaO19.10507 CaO19.2990 | Candida albicans (strain SC5314 / ATCC MYA-2876) (Yeast) | 438 |
| spathulenol | XP_713486.1 | P43063 | CDK1_CANAL | Cyclin-dependent kinase 1 (CDK1) (EC 2.7.11.22) (Cell division control protein 28) (Cell division protein kinase 2) | CDC28 CDK1 CAALFM_CR06050WA CaO19.11337 CaO19.3856 | Candida albicans (strain SC5314 / ATCC MYA-2876) (Yeast) | 317 |
| spathulenol | XP_715542.2 | Q5A1D3 | ERK1_CANAL | Extracellular signal-regulated kinase 1 (ERK1) (EC 2.7.11.24) (MAP kinase 1) (MAPK 1) | CEK1 ERK1 CAALFM_C406480CA CaO19.10404 CaO19.2886 | Candida albicans (strain SC5314 / ATCC MYA-2876) (Yeast) | 421 |
| spathulenol | XP_718680.1 | Q5AAG6 | MKC1_CANAL | Mitogen-activated protein kinase MKC1 (MAP kinase MKC1) (EC 2.7.11.24) | MKC1 CAALFM_CR00120CA CaO19.7523 | Candida albicans (strain SC5314 / ATCC MYA-2876) (Yeast) | 509 |
| spathulenol | XP_719855.1 | Q5ADT3 | Q5ADT3_CANAL | NADP-dependent oxidoreductase domain-containing protein | CAALFM_C307330WA orf19.14050 | Candida albicans (strain SC5314 / ATCC MYA-2876) (Yeast) | 289 |
| spathulenol | XP_719854.1 | Q5ADT4 | Q5ADT4_CANAL | Glycerol 2-dehydrogenase (NADP(+)) | GCY1 CAALFM_C307340WA orf19.14049 | Candida albicans (strain SC5314 / ATCC MYA-2876) (Yeast) | 295 |
| spathulenol | XP_721535.1 | Q5AI15 | PABP_CANAL | Polyadenylate-binding protein, cytoplasmic and nuclear (PABP) (Poly(A)-binding protein) (Polyadenylate tail-binding protein) | PAB1 CAALFM_C103370WA CaO19.10555 CaO19.3037 | Candida albicans (strain SC5314 / ATCC MYA-2876) (Yeast) | 629 |
| spathulenol | XP_721451.2 | Q5AIA1 | EXG2_CANAL | Glucan 1,3-beta-glucosidase 2 (EC 3.2.1.58) (Exo-1,3-beta-glucanase 2) | EXG2 CAALFM_C102630CA CaO19.10469 CaO19.2952 | Candida albicans (strain SC5314 / ATCC MYA-2876) (Yeast) | 479 |

| | | | | | | | |
|---|---|---|---|---|---|---|---|
| spathulenol | XP_723574.1 | Q5AP71 | Q5AP71_CANAL | cAMP-dependent protein kinase (EC 2.7.11.11) | TPK1 CAALFM_C110220CA orf19.12357 | Candida albicans (strain SC5314 / ATCC MYA-2876) (Yeast) | 405 |
| spathulenol | XP_721016.1 | Q92207 | HOG1_CANAL | Mitogen-activated protein kinase HOG1 (MAP kinase HOG1) (EC 2.7.11.24) | HOG1 CAALFM_C203330CA CaO19.8514 CaO19.895 | Candida albicans (strain SC5314 / ATCC MYA-2876) (Yeast) | 377 |
