# Supplementary material for: Quadratic Concentration–Response Modeling and Molecular Docking of Mespilodaphne quixos (Lam.) Rohwer Essential Oil Against Candida albicans
Source: Molecules. 2026 Jun 1;31(11):1891. doi: 10.3390/molecules31111891 (PMC13257838; doi:10.3390/molecules31111891)
Supplement: Supplementary file 1 [file molecules-31-01891-s001.zip › Supplementary_material S2.pdf]

Quadratic Concentration-Response Modeling and Molecular Docking of *Mespilodaphne quixos* (Lam.) Rohwer Essential Oil Against *Candida albicans*

Yasiel Arteaga-Crespo, Yudel García-Quintana, Yendrek Velásquez-López, Matteo Radice, Mariana Magdalena Conforme-García, Jannys Lizeth Rivera-Barreto, José Blanco-Salas, Reinier Abreu-Naranjo*

* Correspondence: rabreu@uea.edu.ec

This supplementary material provides the molecular docking protocol validation by redocking the native ligands of the selected crystallographic structures. The redocking procedure was performed for *exo*-*β*-(1,3)-glucanase (PDB ID: 1EQC), Δ(14)-sterol reductase (PDB ID: 4QUV), and 14-*α*-demethylase (PDB ID: 5TZ1). The agreement between the crystallographic ligand poses and the redocked poses was evaluated using root mean square deviation (RMSD), with values below 3 Å considered acceptable for validating the docking protocol.

Supplementary Material S2: Validation of the molecular docking protocol by redocking native ligands of 1EQC, 4QUV, and 5TZ1.

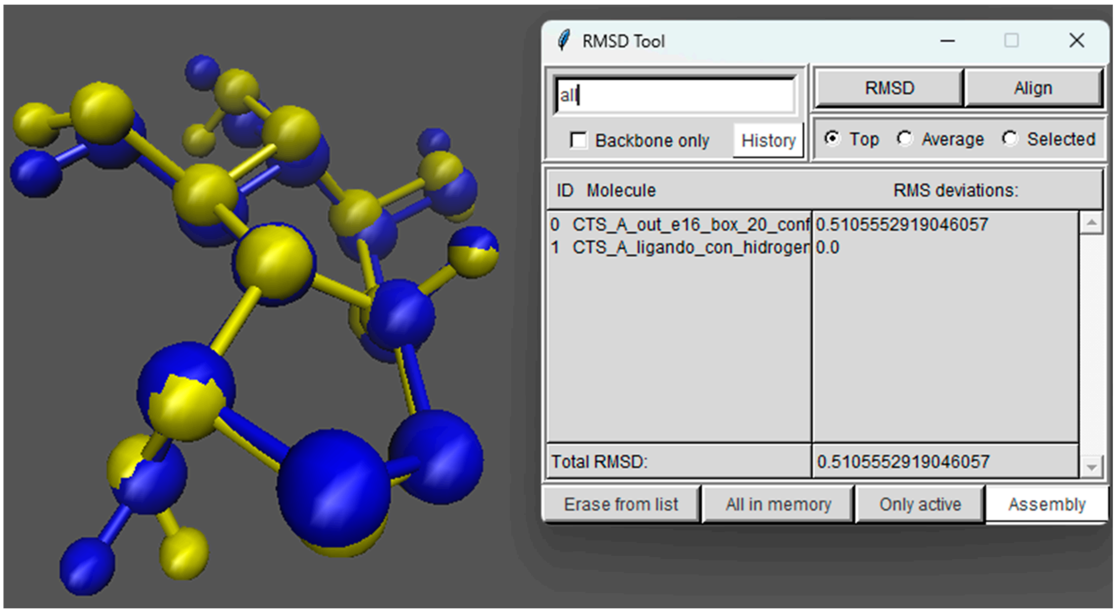

Figure S1. Redocking validation of castanospermine (CTS) in *exo*-*β*-(1,3)-glucanase (PDB ID: 1EQC). The superposition of the native ligand and the redocked pose showed an RMSD of 0.51 Å, indicating adequate reproduction of the crystallographic binding pose.

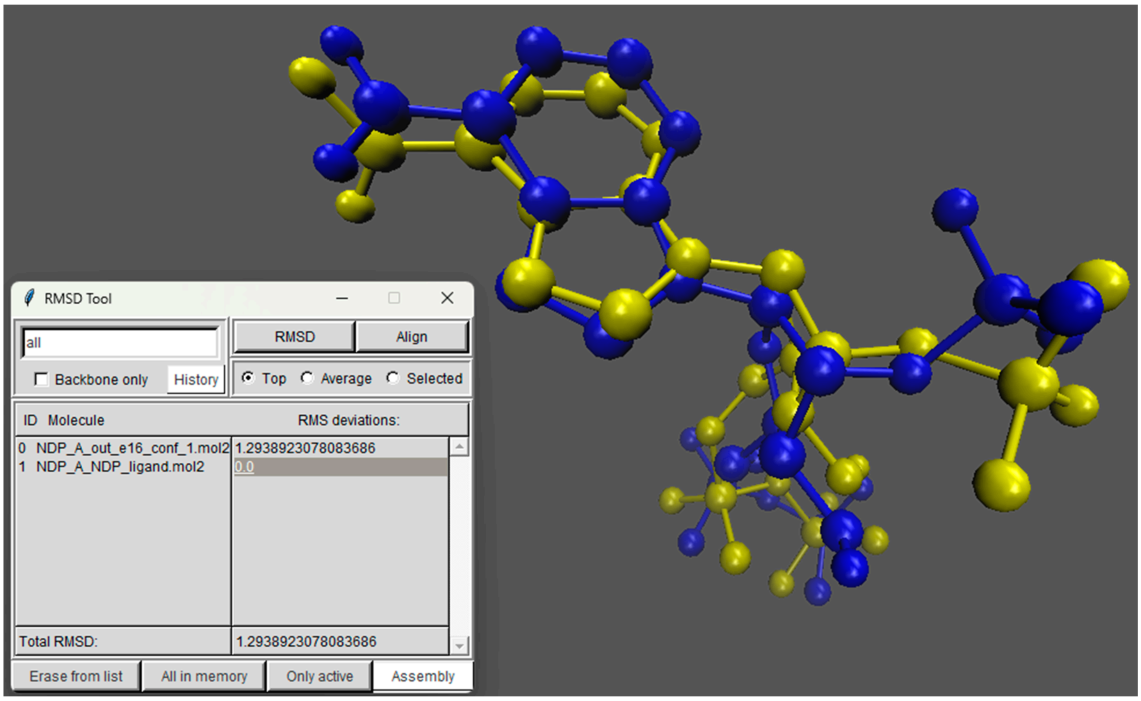

Figure S2. Redocking validation of NADPH dihydro-nicotinamide-adenine-dinucleotide phosphate (NDP) in Δ(14)-sterol reductase (PDB ID: 4QUV). The superposition of the native ligand and the redocked pose showed an RMSD of 1.29 Å, supporting the reliability of the docking protocol.

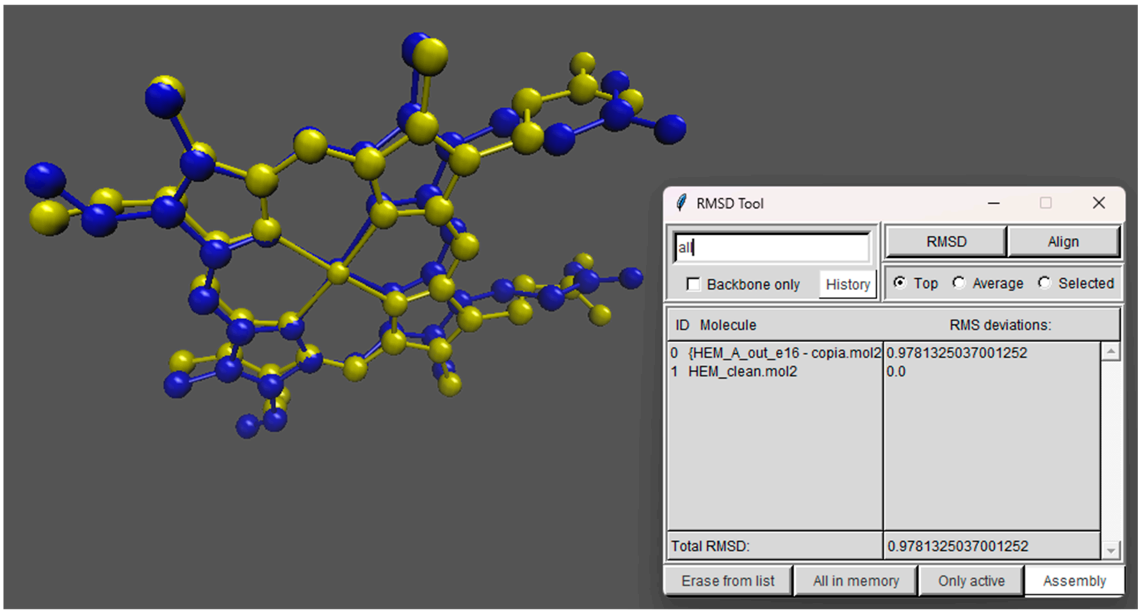

Figure S3. Redocking validation of protoporphyrin IX containing Fe (HEM) in 14-α-demethylase (PDB ID: 5TZ1). The superposition of the native ligand and the redocked pose showed an RMSD of 0.97 Å, indicating adequate redocking performance.
